# Utilizing a Dynamical Description of IspH to Aid in the Development of Novel Antimicrobial Drugs

**DOI:** 10.1371/journal.pcbi.1003395

**Published:** 2013-12-19

**Authors:** Patrick G. Blachly, César A. F. de Oliveira, Sarah L. Williams, J. Andrew McCammon

**Affiliations:** 1 Department of Chemistry & Biochemistry, University of California San Diego, La Jolla, California, United States of America; 2 Howard Hughes Medical Institute, University of California San Diego, La Jolla, California, United States of America; 3 Department of Pharmacology, University of California San Diego, La Jolla, California, United States of America; University of Houston, United States of America

## Abstract

The nonmevalonate pathway is responsible for isoprenoid production in microbes, including *H. pylori*, *M. tuberculosis* and *P. falciparum*, but is nonexistent in humans, thus providing a desirable route for antibacterial and antimalarial drug discovery. We coordinate a structural study of IspH, a [4Fe-4S] protein responsible for converting HMBPP to IPP and DMAPP in the ultimate step in the nonmevalonate pathway. By performing accelerated molecular dynamics simulations on both substrate-free and HMBPP-bound [Fe_4_S_4_]^2+^ IspH, we elucidate how substrate binding alters the dynamics of the protein. Using principal component analysis, we note that while substrate-free IspH samples various open and closed conformations, the closed conformation observed experimentally for HMBPP-bound IspH is inaccessible in the absence of HMBPP. In contrast, simulations with HMBPP bound are restricted from accessing the open states sampled by the substrate-free simulations. Further investigation of the substrate-free simulations reveals large fluctuations in the HMBPP binding pocket, as well as allosteric pocket openings – both of which are achieved through the hinge motions of the individual domains in IspH. Coupling these findings with solvent mapping and various structural analyses reveals alternative druggable sites that may be exploited in future drug design efforts.

## Author Summary

Drug resistance has recently entered into media conversations through the lens of MRSA (methicillin-resistant *Staphylococcus aureus*) infections, but conventional therapies are also failing to address resistance in cases of malaria and other bacterial infections, such as tuberculosis. To address these problems, we must develop new antibacterial and antimalarial medications. Our research focuses on understanding the structure and dynamics of IspH, an enzyme whose function is necessary for the survival of most bacteria and malaria-causing protozoans. Using computer simulations, we track how the structure of IspH changes in the presence and absence of its natural substrate. By inspecting the pockets that form in the normal motions of IspH, we propose a couple new routes by which new molecules may be developed to disrupt the function of IspH. It is our hope that these structural studies may be precursors to the development of novel therapies that may add to our current arsenal against bacterial and malarial infections.

## Introduction

In the past couple decades, antimicrobial drug resistance has risen dramatically and greatly hampered the efficacy of currently available therapies for bacterial and malarial infections [1]–[9]. Whereas (multiple-)drug-resistant bacterial infections are a ubiquitous problem, affecting both the Western world and developing nations, the burdens of malaria fall disproportionately on the poorest regions of the world, with over 219 millions cases and 666,000 deaths reported in 2010 [3]. Beyond the common problems associated with decreased lifetimes for drug efficacy due to rapid development of resistance [1], [2], [5], [6], [9], advances in the fight against bacterial and malarial infections have also been plagued by diminished attention from major pharmaceutical companies toward the development of new therapies and drugs [4], [5], [10]. Consequently, there is urgent need for the development of new drugs with novel modes of action, for administration either independently or in combination with established regimen, both to combat bacterial and malarial infections, as well as to address the propensity of each for rapidly developing drug resistance [1], [4], [6], [7], [9].

**Figure 1:**
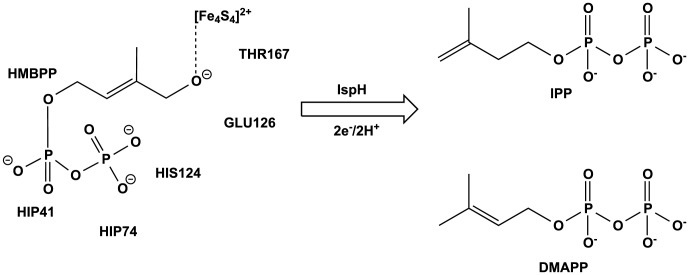
Reductive dehydroxylation of HMBPP affords the isoprenoid precursors, IPP and DMAPP. The relative positions of active site residues suggested to play a role in either substrate binding or catalysis are labeled.

The nonmevalonate pathway for isoprenoid biosynthesis has recently been revealed as a novel target for both antibacterial and antimalarial drugs. Isoprenoids comprise essential metabolites derived from the 5-carbon biomolecules, isopentenyl diphosphate (IPP) and dimethylallyl diphosphate (DMAPP, Figure 1), examples of which include sterols that provide structural support to membranes, chlorophylls used in photosynthesis, and quinones that participate in electron transport chains [11]–[14]. In contrast, animals acquire IPP and DMAPP in a distinctive manner via a mevalonate-dependent pathway. Given this metabolic difference, the proteins involved in the nonmevalonate pathway provide novel targets for the development of antibacterial and antimalarial drugs that are both broadly specific to pathogenic species such as *H. pylori*, *M. tuberculosis* and *P. falciparum* and without known human analogs [15]–[18].

The ultimate step in the nonmevalonate pathway is the generation of IPP and DMAPP through a 2-electron reductive dehydroxylation of (E)-1-hydroxy-2-methyl-but-2-enyl pyrophosphate (HMBPP) by IspH, a [4Fe-4S] protein (Figure 1) [11], [19]–[21]. The catalytic mechanism of IspH has been a topic of great debate, largely due to uncertainties introduced by the iron-sulfur cluster [18], [22]. Initial structures of IspH from *Aquifex aeolicus* [23] and *Escherichia coli* [24] solved by X-ray crystallography resemble cloverleaves and comprise three sequentially different domains with pseudo-C3 symmetry, each tethered to a [Fe_3_S_4_]^+^ cluster via a conserved cysteine residue. The *A. aeolicus* [Fe_3_S_4_]^+^ IspH structure (PDB ID: 3DNF; henceforth referred to as [Fe_3_S_4_]^+^_(open, substrate-free)_ IspH) assumes an open conformation, with a 10×20 Å cavity where the HMBPP molecule is expected to bind at the cluster [23]. In contrast to the *A. aeolicus* crystal structure, the [Fe_3_S_4_]^+^ *E. coli* counterpart is closed around an inorganic diphosphate molecule (PP*_i_*) that sits in the vicinity of the centrally located [Fe_3_S_4_]^+^ cluster. Various conserved polar and charged residues, including Glu-126, Thr-167, Asn-227, His-41, His-74, His-124, Ser-225, Ser-226 and Ser-269 (*E. coli* numbering scheme), coordinate the PP*_i_* molecule, likely via hydrogen bonding or salt bridge interactions [24]. The orientations of these conserved residues in the *E. coli* structure are distinct from their *A. aeolicus* counterparts due to a tilt of a single domain that enables co-localization of charged and polar residues around the PP_i_ in the case of the former.

**Figure 2:**
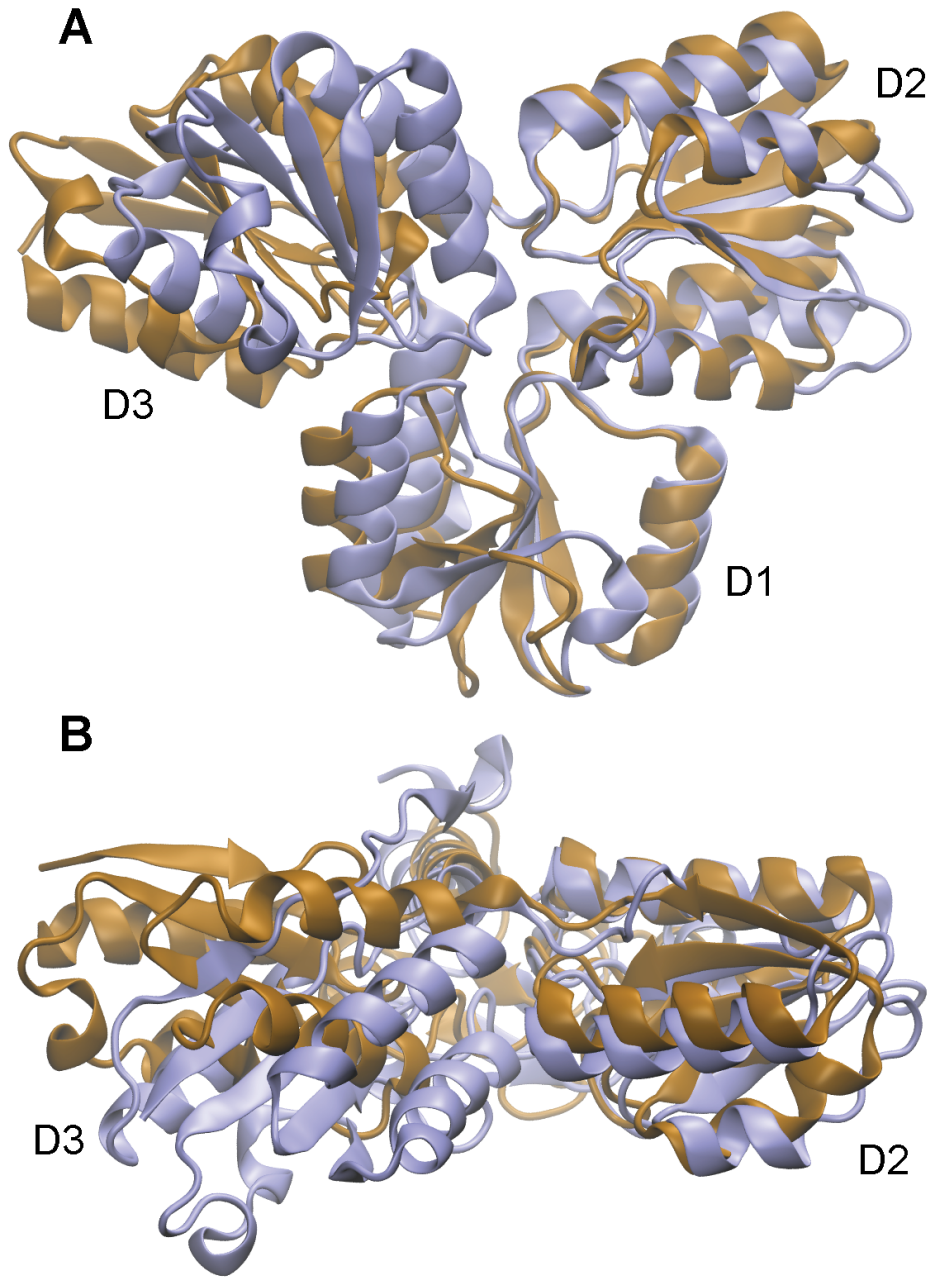
Superposition of [Fe_3_S_4_]^+^_(open, substrate-free)_ (bronze, [23]) and [Fe_4_S_4_]^2+^_(closed, HMBPP-bound)_ (purple, [28]) IspH crystal structures, viewed (A) head-on toward the binding site and (B) from a top-view highlighting the domain tilt of D3.

While results from electron paramagnetic resonance (EPR) spectroscopy have shown [Fe_3_S_4_]^+^ IspH to be catalytically active [25], reconstituted IspH displays EPR and Mossbauer signatures of a [Fe_4_S_4_]^2+^ cluster [26], [27]. Groll and co-workers provide further support for the catalytically relevant form of IspH containing a [Fe_4_S_4_]^2+^ cluster with their work in crystallizing IspH in the presence of its substrate, HMBPP. This HMBPP-bound crystal structure (PDB ID: 3KE8, henceforth referred to as [Fe_4_S_4_]^2+^_(closed, HMBPP-bound)_ IspH) assumes a closed conformation having a domain tilt similar to that of the [Fe_3_S_4_]^+^ *E. coli* structure, with HMBPP bound via its terminal hydroxyl moiety to an unliganded iron of a [Fe_4_S_4_]^2+^ cluster (Figure 2) [28]. The coordination sphere of the HMBPP ligand is virtually identical to the inorganic diphosphate molecule, while its terminal hydroxyl moiety interacts with Glu-126, Thr-167 (*E. coli* numbering) and an ordered water molecule to make a hydrogen bond network that is proposed to facilitate proton transfer during catalysis [28]. While these structural data provide a good picture of the [Fe_4_S_4_]^2+^ IspH structure with HMBPP bound, the structure of the 4Fe-form in the absence of substrate, as well as a detailed understanding of how IspH changes conformation upon ligand binding, are not fully understood.

Drawing from insight gained from the aforementioned structural work, as well as various spectroscopic and mutational studies, multiple groups have contributed to drug discovery efforts on the IspH target [29]–[34]. To the best of our knowledge, IspH inhibitor development has fallen under two classes: (1) HMBPP analogues [29]–[31] and (2) pyridine or alkenyl/alkynyl diphosphates and bisphosphonates [32]–[34]. In the case of HMBPP analogues, inhibitor binding emulates the natural substrate, while leveraging improved interactions with the Fe-site (*e.g.* binding of a thiol instead of an alcohol) [30], [31]. Alternatively, Oldfield and co-workers have created novel inhibitors of IspH by utilizing olefinic and pyridine groups to form π/σ “metallacycle” complexes and η^1^-complexes, respectively, coupling these metal binding groups to phosphate skeletons that preserve the hydrogen bond and salt bridge interactions present in IspH-HMBPP complexation [32]–[34]. These initial drug discovery efforts may be enhanced, both in terms of finding new lead compounds and developing already discovered leads, by obtaining a better description of the IspH binding pocket and possible allosteric sites that may be targeted.

Given that there exists no high-resolution structural data for substrate-free, [Fe_4_S_4_]^2+^ IspH, this work employs accelerated molecular dynamics (aMD) simulations to describe the dominant conformations available to IspH having a fourth iron atom in the absence of HMBPP. Characterization of these dominant conformations reveals an expanded binding pocket and allosteric sites that may be targeted with future rational drug design efforts. Additional attention is directed toward understanding how IspH dynamics are altered upon ligand binding, allowing us to propose a mechanism for how IspH-HMBPP complexation is achieved.

## Results

### RMSD and visual analyses of aMD simulations of open, substrate-free IspH

**Figure 3:**
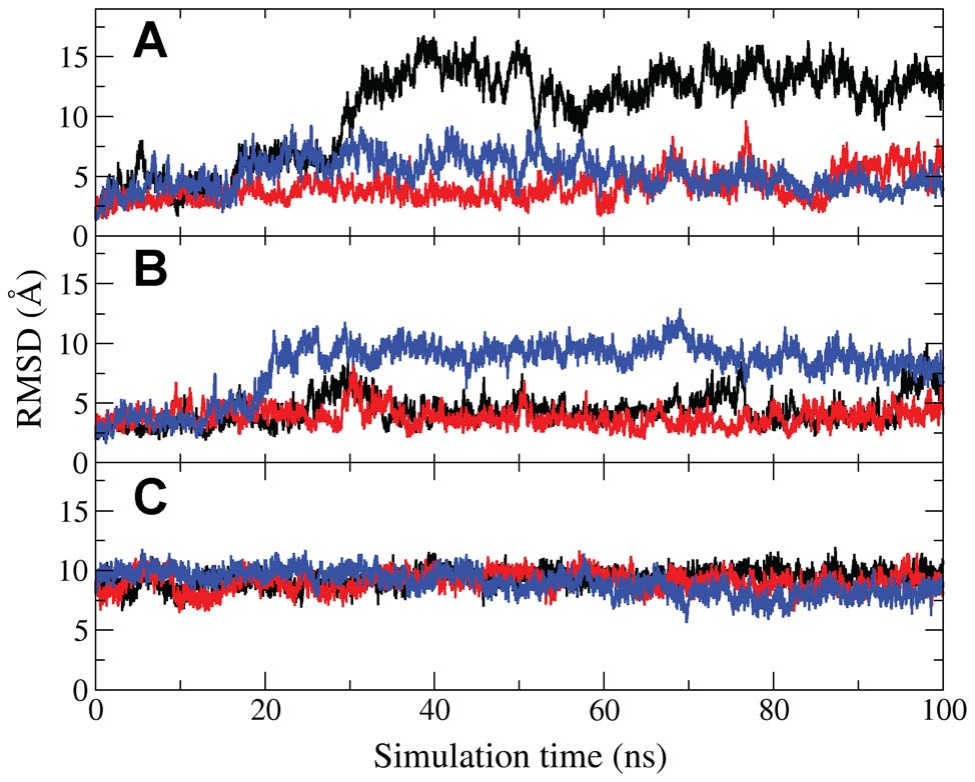
Plots of RMSD relative to the [Fe_3_S_4_]^+^_(open, substrate-free)_ crystal structure over the course of 3×100 ns aMD simulations of (A) [Fe_4_S_4_]^2+^_(open,substrate-free)_, (B) [Fe_4_S_4_]^2+^/HMBPP_(open,docked)_, and (C) [Fe_4_S_4_]^2+^/HMBPP_(closed)_ IspH.

Consistent with the nomenclature used by Gräwert, *et al.* [28], descriptions of IspH from this point forward will use the nomenclature D1, D2 and D3 to describe the domains containing residues 14–96; 97–193; and 194–281, 1–13, respectively (*A. aeolicus* numbering, Figure 2). We perform 3×100 ns aMD simulations of [Fe_4_S_4_]^2+^_(open, substrate-free)_ IspH, starting from the *A. aeolicus* crystal structure with a fourth iron modeled into the cluster, as described in the Methods. All trajectories are aligned to the [Fe_3_S_4_]^+^_(open, substrate-free)_ IspH crystal structure by the backbone atoms of all D1 residues, since these residues display significantly lower fluctuation throughout the simulation than those in D2 and D3 [23]. The root-mean-square deviation (RMSD) for the backbone atoms of all residues after alignment is given in Figure 3a. From this RMSD analysis, it is apparent that each independent trajectory samples conformational space differently. The large changes in RMSD correspond to opening and closing motions of the D2 and D3 domains, providing a more dynamic description of the [Fe_4_S_4_]^2+^_(open, substrate-free)_ state than is acquired from a static X-ray structure. While all three simulations extensively sample conformational space near the [Fe_3_S_4_]^+^_(open, substrate-free)_ IspH crystal structure for the first ∼20 ns of the simulation, one simulation diverges from this experimental reference, implying that other distinctive, low-energy conformational states exist for substrate-free IspH.

### Docking of HMBPP to open IspH

Using Schrodinger's Glide program [35]–[37], we dock HMBPP to the unique iron site in IspH. Docked poses are filtered applying knowledge from experiment that the terminal alkoxide/alcohol group of HMBPP directly chelates the apical Fe site [26], [28], [38], [39]. The docked pose used in our MD studies is found by constraining the position of the terminal alkoxide moiety to within a 2.5 Å radius of the apical iron. While the orientation of the PP*_i_* moiety in our docked pose differs from the [Fe_4_S_4_]^2+^_(closed, HMBPP-bound)_ IspH crystal structure (3KE8) [28], it is worth mentioning that the cyclic structure of HMBPP observed in the crystal structure likely results from “induced fit” effects, with polar and charged groups closing around the PP*_i_* moiety. Given these effects are absent from our docking procedure, we use the Glide geometry as a starting point for elucidating how open, substrate-free IspH responds to the formation of an encounter complex with HMBPP bound to its unliganded Fe.

### RMSD and visual analyses of aMD simulations of apo-IspH with HMBPP docked into the active site

Similar to the [Fe_4_S_4_]^2+^_(open, substrate-free)_ simulations, three independent, 100 ns aMD simulations of HMBPP docked into the open, [Fe_4_S_4_]^2+^-IspH structure (henceforth referred to as [Fe_4_S_4_]^2+^/HMBPP_(open, docked)_) are aligned to the [Fe_3_S_4_]^+^_(open, substrate-free)_ IspH crystal structure, with the RMSD of all backbone atoms to the crystal structure given in Figure 3b. Both seeds one and three (Figure 3b, black and blue, respectively) approach an RMSD of ∼8–10 Å, with respect to the crystal structure. This jump occurs rapidly for seed three (in the first 20 ns of simulation), while seed one only appears to approach this level in the last 10 ns of simulation. This shift from the [Fe_3_S_4_]^+^_(open, substrate-free)_ IspH crystal structure results from the closing of D2 and D3 around the docked HMBPP, matching the conformation assumed by the [Fe_4_S_4_]^2+^_(closed, HMBPP-bound)_ IspH crystal structure (Figure S1).

**Figure 4:**
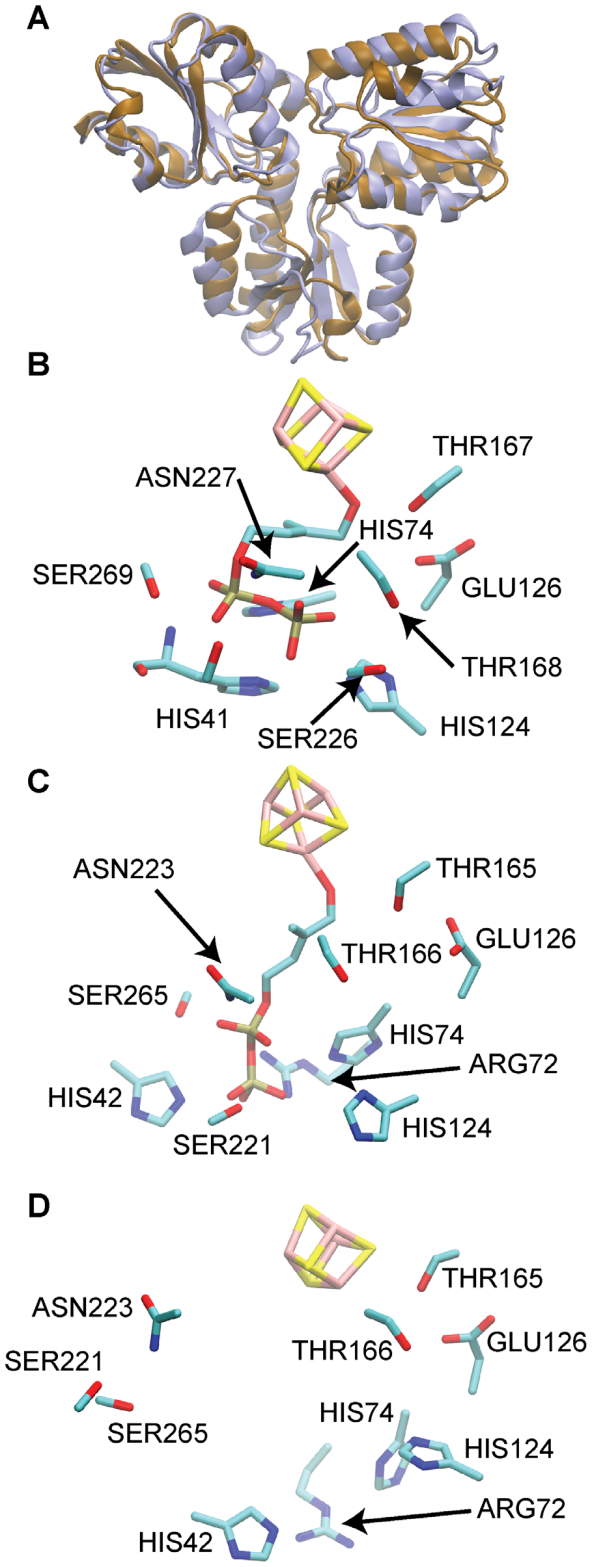
Comparison of HMBPP-bound IspH structures from experiment and simulation. (A) shows the superposition of the [Fe_4_S_4_]^2+^_(closed, HMBPP-bound)_ IspH crystal structure (bronze, [28]) and a representative structure from the dominant closed cluster from [Fe_4_S_4_]^2+^/HMBPP_(open,docked)_ aMD simulations (purple). (B) and (C) correspond to the active site microenvironments of the crystal structure and the representative closed structure from aMD, respectively, while (D) illustrates the positions of the residues present in (B) and (C) in the [Fe_3_S_4_]^+^_(open, substrate-free)_ crystal structure [23].

To gain insight into the dominant conformations sampled by these [Fe_4_S_4_]^2+^/HMBPP_(open, docked)_ simulations, we cluster the frames of each trajectory according to pairwise RMSD comparing C_α_ atoms, as described in the Methods. The dominant cluster (58%) corresponds to an open conformation, similar to the [Fe_3_S_4_]^+^_(open, substrate-free)_ IspH crystal structure [28]. The second most populated cluster (18%) contains closed structures resembling the [Fe_4_S_4_]^2+^_(closed, HMBPP-bound)_ IspH crystal structure (Figure 4). When considering the structures in this closed cluster, it is notable that the ligand does not form a ring structure consistent with its pose in the crystal structure. Nevertheless, the closing of the D2 and D3 domains around the substrate is consistent with the [Fe_4_S_4_]^2+^_(closed, HMBPP-bound)_ experimental reference [28]. A more detailed inspection of the HMBPP environment in a representative structure from this closed cluster reveals the three key active site histidines, as well as the conserved Thr-165, Thr-166, Glu-126, Ser-221, Asn-223 and Ser-265 forming contacts with HMBPP that appear identical to those seen in the [Fe_4_S_4_]^2+^_(closed, HMBPP-bound)_ IspH crystal structure (Figure 4b,c). While Glu-126 and Thr-167 are co-localized with the iron-sulfur cluster in the active site of [Fe_4_S_4_]^2+^_(open, substrate-free)_ IspH (Figure 4d), the other contacts mentioned are unique to substrate-bound IspH, as seen in the [Fe_4_S_4_]^2+^_(closed, HMBPP-bound)_ crystal structure (Figure 4b). These findings demonstrate that aMD simulations have effectively captured the closing of loops from D2 and D3 around HMBPP—confirming earlier hypotheses for how conformational change occurs upon substrate binding [28].

Inconsistent with the RMSD results for seed three, both seeds one and two (black and red, Figure 3b) are trapped in a basin near the [Fe_3_S_4_]^+^_(open, substrate-free)_ IspH crystal structure for a majority of their respective simulations. These differing trajectories arise, in part, because the residues in D3 that coordinate the PP*_i_* of HMBPP in seeds one and two do not coordinate the bound HMBPP. For instance, the side chain of Ser-265 in seed two does not extend inward toward the bound substrate, instead interacting with loop residues at the interfaces of D3 with D1 (Phe-12, Asn-43 and Thr-266). The local conformations of these residues are more consistent with those observed in [Fe_4_S_4_]^2+^_(open, substrate-free)_ simulations of IspH. Coupled with the observed closing event in seed three, these findings demonstrate the presence of a barrier between the open and closed states, requiring the intramolecular interactions present in the substrate-free state to break in order to form interactions with bound HMBPP.

### Simulations of IspH in complex with HMBPP started from a closed structure

The second most populated cluster from [Fe_4_S_4_]^2+^/HMBPP_(open, docked)_ IspH simulations, which corresponds to the most populated closed conformation, is used as a starting point for three additional 100 ns aMD simulations (henceforth referred to as [Fe_4_S_4_]^2+^/HMBPP_(closed)_ simulations). Plots of the computed RMSD with respect to the [Fe_3_S_4_]^+^_(open, substrate-free)_ IspH crystal structure for these simulations are marked by their lack of change, not deviating more than ∼3 Å from the closed conformations sampled in [Fe_4_S_4_]^2+^/HMBPP_(open, docked)_ simulations (Figure 3c, Figure S1). Similar to what is seen in the [Fe_4_S_4_]^2+^/HMBPP_(open, docked)_ aMD simulations, we note that HMBPP never fully reaches its ring conformation seen crystallographically [28]. From these simulations, it is evident that substrate-bound IspH, once folded around HMBPP, has less conformational space accessible to it and does not access open states.

### Assessing sampling using principal component analysis

In constructing principal component (PC) space from all [Fe_4_S_4_]^2+^_(open, substrate-free)_ and [Fe_4_S_4_]^2+^/HMBPP_(open, docked)_ simulations, as described in the Methods, we observe that the first two principal components account for 83% of the variance. Using Bio3D [40], the motions that correspond to movement along PC1 and PC2 are visualized (Figure S2) and are shown to correspond to opening and closing motions achieved through the hinge-like properties of the loops that connect D3 to D1 and D2 and D2 to D1 and D3, as suggested by Groll and co-workers [28].

**Figure 5:**
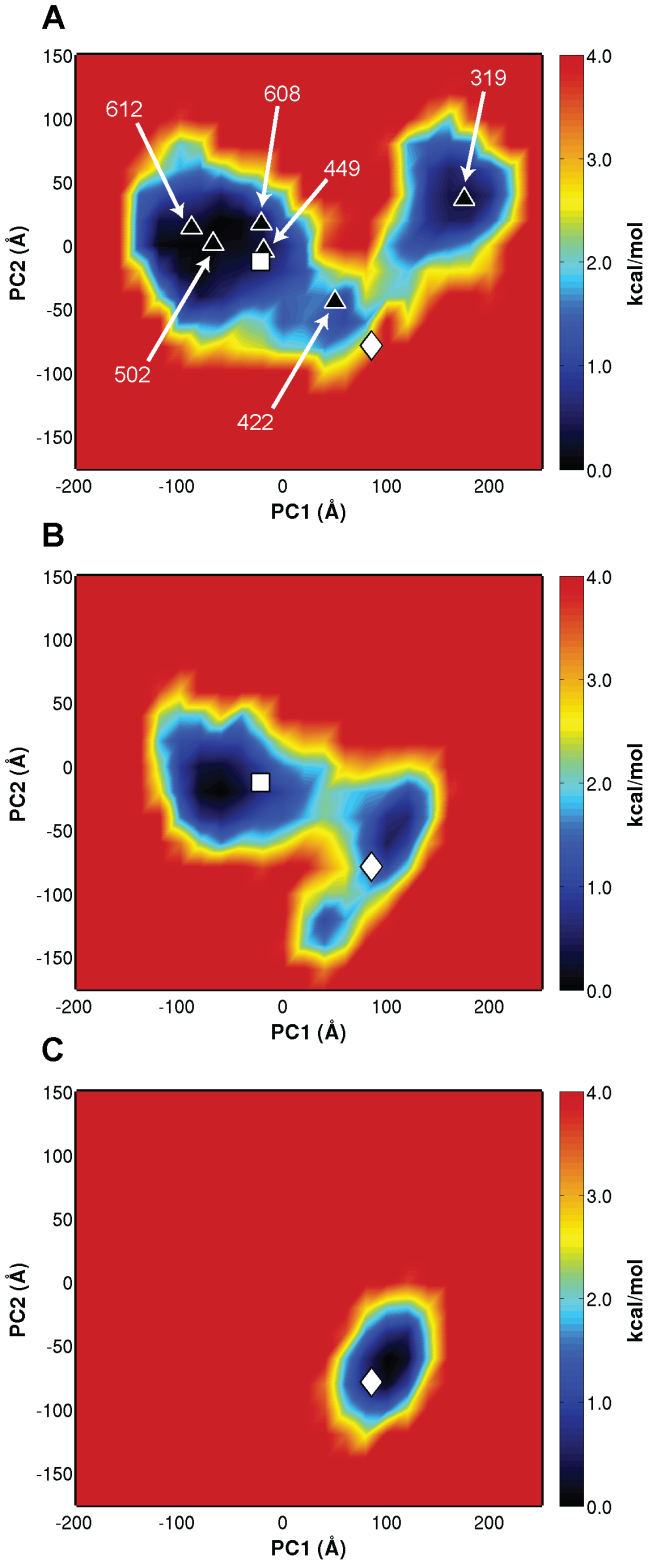
Projections of (A) [Fe_4_S_4_]^2+^_(open,substrate-free)_, (B) [Fe_4_S_4_]^2+^/HMBPP_(open,docked)_, and (C) [Fe_4_S_4_]^2+^/HMBPP_(closed)_ trajectories onto principal component (PC) space constructed from [Fe_4_S_4_]^2+^_(open,substrate-free)_ and [Fe_4_S_4_]^2+^/HMBPP_(open,docked)_ aMD simulations. Crystal structures corresponding to [Fe_3_S_4_]^+^_(open, substrate-free)_ (white square, PDB ID: 3DNF) and [Fe_4_S_4_]^2+^_(closed, HMBPP-bound)_ IspH (white diamond, PDB ID: 3KE8) are also projected onto PC space [23], [28]. Numbers in (A) correspond to POVME volumes (Å^3^), as described in the text.

All simulations ([Fe_4_S_4_]^2+^_(open,substrate-free)_, [Fe_4_S_4_]^2+^/HMBPP_(open,docked)_, and [Fe_4_S_4_]^2+^/HMBPP_(closed)_), as well as the coordinates from the [Fe_3_S_4_]^+^_(open, substrate-free)_ and the [Fe_4_S_4_]^2+^_(closed, HMBPP-bound)_ IspH crystal structures, are projected onto the PC space to assess how the simulations sample configuration space with respect to the crystal structures within this coordinate system (Figure 5). Viewing these projections, it is clear that the [Fe_4_S_4_]^2+^_(open,substrate-free)_ simulations (Figure 5a) sample significantly greater conformational space than the [Fe_4_S_4_]^2+^/HMBPP_(open,docked)_ and [Fe_4_S_4_]^2+^/HMBPP_(closed)_ simulations (Figure 5b,c). While other local minima are present, the [Fe_4_S_4_]^2+^_(open,substrate-free)_ simulations sample energy wells near both the open (PDB ID: 3DNF) and closed (PDB ID: 3KE8) crystal structures along PC1 but do not overlap with the latter, HMBPP-bound crystal structure. This finding suggests that the precise closing motions that accompany ligand binding are absent without HMBPP bound to IspH, despite the intrinsic ability of [Fe_4_S_4_]^2+^_(open,substrate-free)_ IspH to sample alternative closed states.

Volume analysis of the states sampled in the [Fe_4_S_4_]^2+^_(open,substrate-free)_ IspH simulations demonstrates the extent to which various open and closed states are sampled within this PC framework. Using the Pocket Volume MEasurer (POVME) program [41], the volumes of representative structures from the clusters generated from [Fe_4_S_4_]^2+^_(open,substrate-free)_ aMD trajectories are obtained and given in Figure 5a. Using this algorithm, it is notable that the [Fe_3_S_4_]^+^_(open, substrate-free)_ IspH crystal structure [23] has a binding pocket volume of 451 Å^3^, whereas the [Fe_4_S_4_]^2+^_(closed, HMBPP-bound)_ IspH crystal structure [28] has a volume of 6 Å^3^ (71 Å^3^, in the absence of HMBPP). Movement along PC1 generally accompanies a decrease in binding pocket size in the [Fe_4_S_4_]^2+^_(open,substrate-free)_ aMD simulations (from 612 Å^3^ at the most negative values of PC1 to 319 Å^3^ at the most positive values, Figure 5a). The characteristics of these different pockets are probed later in this report.

Projection of the [Fe_4_S_4_]^2+^/HMBPP_(open,docked)_ simulations onto PC space reveals a single, clear pathway for the transition between open and closed states (Figure 5b). Three minima are apparent in the projections, one centered near the substrate-free crystal structure and two near the HMBPP-bound crystal structures that differ slightly in the specific contacts made between the protein and ligand. The extent to which bound-HMBPP restricts IspH dynamics is highlighted from the projection of the [Fe_4_S_4_]^2+^/HMBPP_(closed)_ simulations onto PC space. When simulated from a closed conformation, it is clear that bound-HMBPP effectively locks the protein in a closed conformation, unable to access open states—evident by a well present only around the closed, HMBPP-bound IspH crystal structure (Figure 5c).

### RMSF analysis shows a decrease in fluctuation accompanies ligand binding

**Figure 6:**
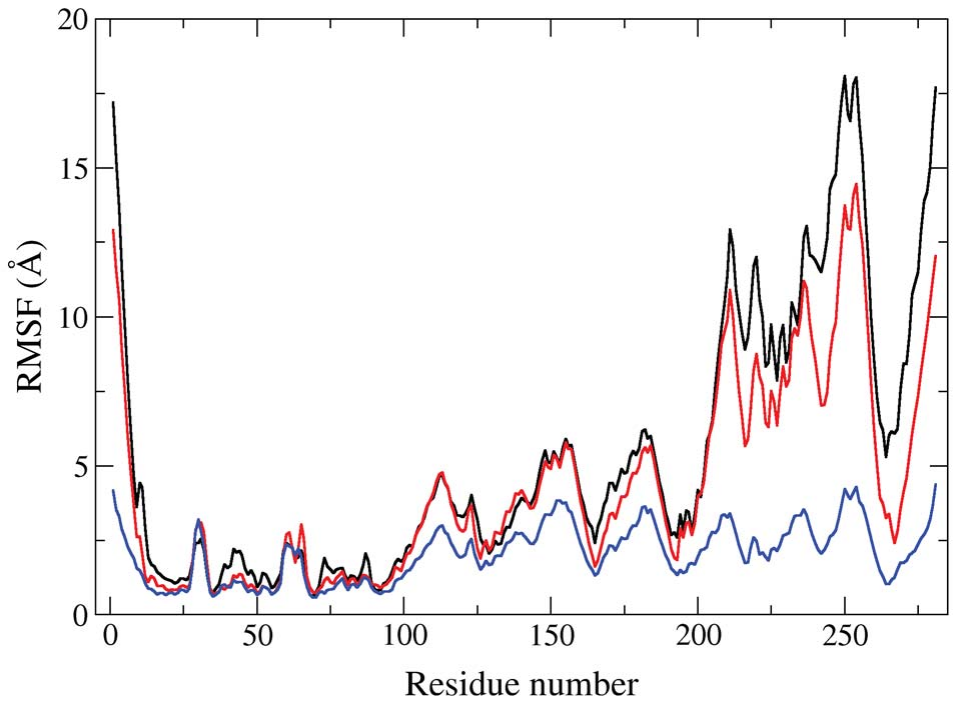
RMSF analysis of [Fe_4_S_4_]^2+^_(open,substrate-free)_ (black), [Fe_4_S_4_]^2+^/HMBPP_(open,docked)_ (red), and [Fe_4_S_4_]^2+^/HMBPP_(closed)_ (blue) aMD simulations.

Combining the three trajectories for each individual system simulated, we performed a root-mean-square fluctuation (RMSF) analysis to quantify the extent to which each residue fluctuates in the different systems (Figure 6). In the case of the [Fe_4_S_4_]^2+^_(open,substrate-free)_ simulations (Figure 6, black curve), the fluctuations in D2 and D3 are slightly greater than what is seen in the [Fe_4_S_4_]^2+^/HMBPP_(open,docked)_ simulations. These fluctuations are abolished when the simulations are started from a closed conformation with HMBPP bound ([Fe_4_S_4_]^2+^/HMBPP_(closed)_ simulations).

### Obtaining an understanding of local phenomena driving conformational change upon HMBPP binding

Changes in various peptide dihedral angles (phi, psi and chi) typically accompany global changes in protein conformation [42], [43]. In other words, certain dihedral angles may select for specific conformations in proteins [42], [43]. Recently, McClendon *et al.* contributed a method that quantifies differences in probability distributions of protein dihedral angles between a reference and altered state of a protein by using an expansion of the Kullback-Leibler (KL) Divergence [42]. This application assigns a value for the “mutual divergence” of each residue—a measure of the extent to which the distributions of dihedral angles differ between the two states. Using the [Fe_4_S_4_]^2+^_(open,substrate-free)_ simulation as a reference, we compute “mutual divergence” values upon substrate binding to IspH using the MutInf suite of programs [42], [44] in an attempt to isolate local changes in protein structure that give rise to globally different conformational ensembles between the [Fe_4_S_4_]^2+^_(open,substrate-free)_ and [Fe_4_S_4_]^2+^/HMBPP_(closed)_ aMD simulations.

**Figure 7:**
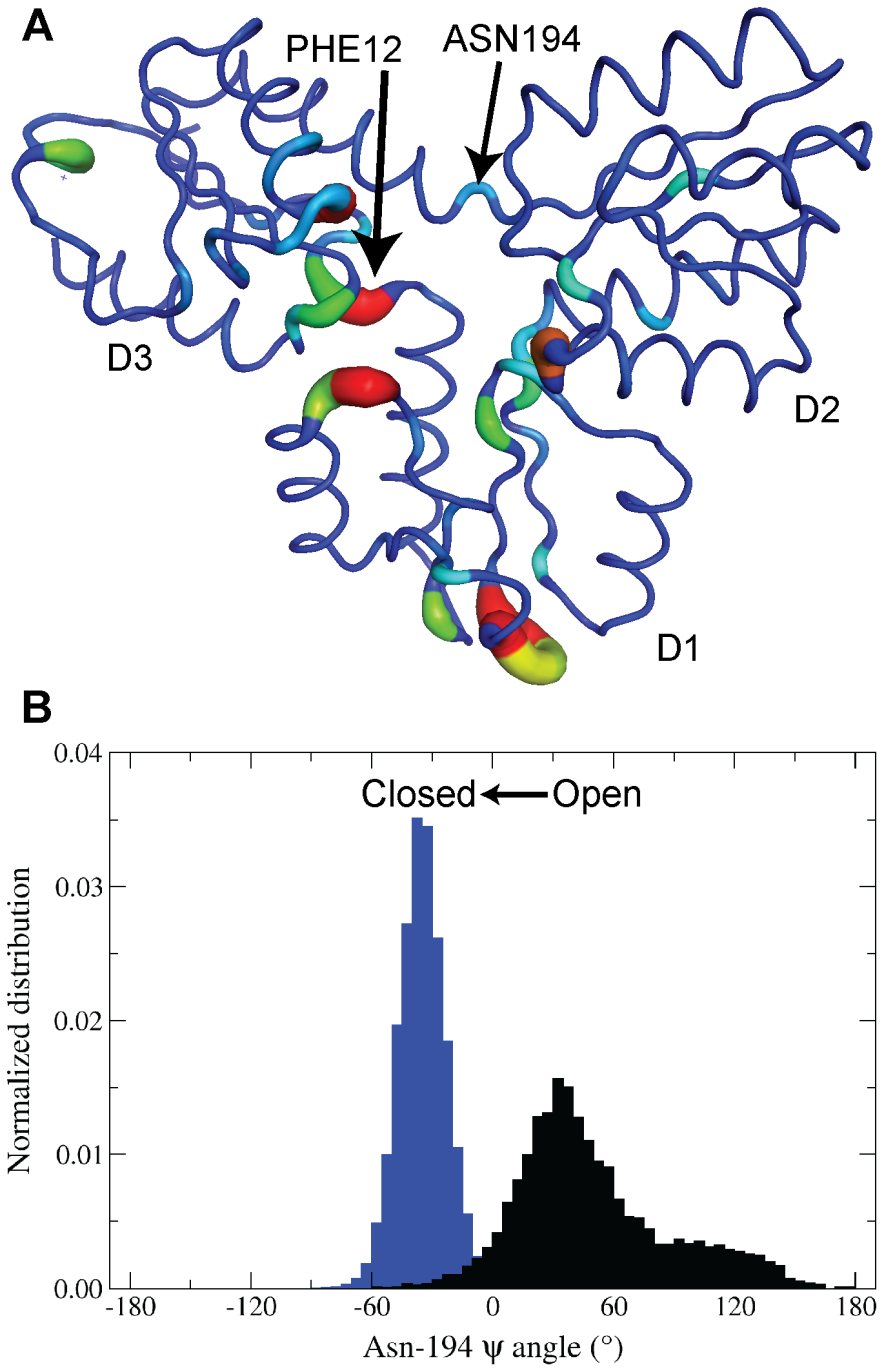
(A) Use of the Kullback-Leibler (KL) divergence to highlight residues with distinct dihedral distributions between [Fe_4_S_4_]^2+^_(open,substrate-free)_ and [Fe_4_S_4_]^2+^/HMBPP_(closed)_ simulations of IspH. Visualization of residues with high “mutual divergence” in the IspH structure, ranging from blue (low) to red (high). (B) Illustration of the different dihedral distributions of the Asn-194 ψ-angles sampled in open (black) and closed (blue) conformations of IspH.

A visual representation of “mutual divergence” values is provided in Figure 7a, with the highest scoring residues shown in Table 1. We present these data together with measures of sequence conservation, computed as Shannon entropy [40], [45], [46], as both these metrics are suggested to highlight residues of functional importance [42], [45]. The link between sequence conservation and functionality is obvious—residues that are highly conserved are usually conserved for some purpose, *e.g.* to bestow certain structural features to a protein or to participate in catalysis. Similarly, residues whose conformations change dramatically upon some natural perturbation to the system, ligand binding in our case, are likely responsible for the functionality of that protein. Consequently, we propose that residues that both are highly conserved and display high “mutual divergence” upon ligand binding are critical to the structure and function of IspH.

**Table 1:** Residues with “mutual divergence” values greater than 1.0 and their respective sequence conservation scores, as computed by Shannon entropy [40], [45], [46].

Residue	Mutual Divergence	Sequence conservation score
GLY11	**1.01**	**0.99**
PHE12	**3.06**	**0.83**
LYS33	1.17	0.31
HIS42	**2.44**	**0.99**
ASN43	**1.26**	**0.95**
PHE63	2.25	0.37
LYS64	1.46	0.00
GLU65	1.35	0.17
GLY66	2.42	0.24
ASP67	5.18	0.18
ARG72	**1.03**	**0.77**
HIS124	**1.79**	**1.00**
ASN223	**2.31**	**1.00**
TRP251	1.16	0.38
SER265	**1.03**	**1.00**

Interestingly, five residues displaying higher levels of “mutual divergence” (Phe-63, Lys-64, Glu-65, Gly-66 and Asp-67) are located in a loop region in D1 and are neither conserved nor directly interacting with bound-HMBPP (Figure 7, Table 1). Arg-72 and His-74 are positioned at the opposite end of this loop region and form hydrogen bonds with the PP*_i_* tail of HMBPP in the [Fe_4_S_4_]^2+^/HMBPP_(closed)_ simulations. From these observations, it can be reasoned that the conformations of Arg-72 and His-74, altered upon HMBPP binding, in turn disrupt the conformations of the residues at the end of the loop.

Most other residues with high “mutual divergence” can be characterized by one of two distinct environments in the protein: either (a) coordinating HMBPP when it is bound (*e.g.* His-42, His-124, Asn-223 and Ser-265); or (b) structurally flanking the thiolates that anchor the [Fe_4_S_4_]^2+^ cluster to the protein (as is the case for Phe-12, which is adjacent to Cys-13).

High mutual divergence is seen for residues that occupy the first coordination shell of HMBPP when it is bound. These residues assume different conformations based upon whether they are coordinating the substrate. For instance, both His-42 and Arg-72 from D1, His-124 from D2 and both Asn-223 and Ser-265 from D3 all assume different main and side chain dihedral angle distributions in the [Fe_4_S_4_]^2+^/HMBPP_(closed)_ state compared to the [Fe_4_S_4_]^2+^_(open,substrate-free)_ state. These differences derive from the reorientation of these residues about the PP*_i_* of HMBPP in order to participate in hydrogen bonds or salt bridges.

The other class of residues with high “mutual divergence” reside adjacent to the thiolates tethered to the [Fe_4_S_4_]^2+^ cluster. Phe-12 exemplifies this finding, in that it maintains altered φ/ψ angle distributions, contingent on whether HMBPP is bound (Figure S3). The case of Phe-12 suggests similar behavior may exist in other thiolate-adjacent residues. In inspecting the dihedral angle distributions of Thr-95 and Asn-194 in the [Fe_4_S_4_]^2+^_(open,substrate-free)_ and [Fe_4_S_4_]^2+^/HMBPP_(closed)_ simulations (both having more modest “mutual divergence” scores of 0.77 and 0.32, respectively; Figure S3), it is evident that while the φ/ψ angle distributions are virtually identical for Thr-95, Asn-194 samples different distributions in HMBPP-free and bound states, much like Phe-12. Unlike Phe-12, however, the φ/ψ angles of Asn-194 are unimodal in the closed simulations, indicating that closed conformations require that Asn-194 maintain certain backbone dihedral angles. Indeed, when the [Fe_4_S_4_]^2+^_(open,substrate-free)_ and [Fe_4_S_4_]^2+^/HMBPP_(closed)_ simulations are combined and clustered together into open and closed conformations, it is clear that Asn-194 samples entirely different psi angles, contingent on whether D2 and D3 are open or closed (Figure 7b).

Whereas the psi angle for Asn-194 in open states contributes to the residue's disordered secondary structure, as computed by STRIDE calculations [47], Asn-194 in all closed states is strictly α-helical with a mean psi angle of −34°. It is clear from these distributions that dihedral angles near −34° select for the closed conformations of IspH and contribute to the helicity of Asn-194, unseen in the open conformations that only exist in the ensemble of states sampled in [Fe_4_S_4_]^2+^_(open,substrate-free)_ simulations.

Moving from the dihedral angle to the global structure of D3, it is evident that the helicity of Asn-194 is achieved via cranking motions that pull the helix, comprised of residues 195 to 207 and anchored by Asn-194, behind the [4Fe-4S] cluster in all closed states. This “crank” motion effectively compresses the D3 domain and also draws inward the loops needed to corral HMBPP into a closed active site. In contrast, Asn-194 samples states with no ordered secondary structure in [Fe_4_S_4_]^2+^_(open,substrate-free)_ IspH simulations, while extended in the open conformation.

### Applying knowledge of the expanded binding pocket from apo simulations to motivate drug discovery efforts

Clustering of the [Fe_4_S_4_]^2+^_(open,substrate-free)_ IspH aMD simulations reveals dominant structures with substrate pockets of differing volumes and chemical environments. Using representative structures from each of the clusters, we investigate the druggability of these different pockets by performing solvent mapping with the FTMAP program [48].

**Figure 8:**
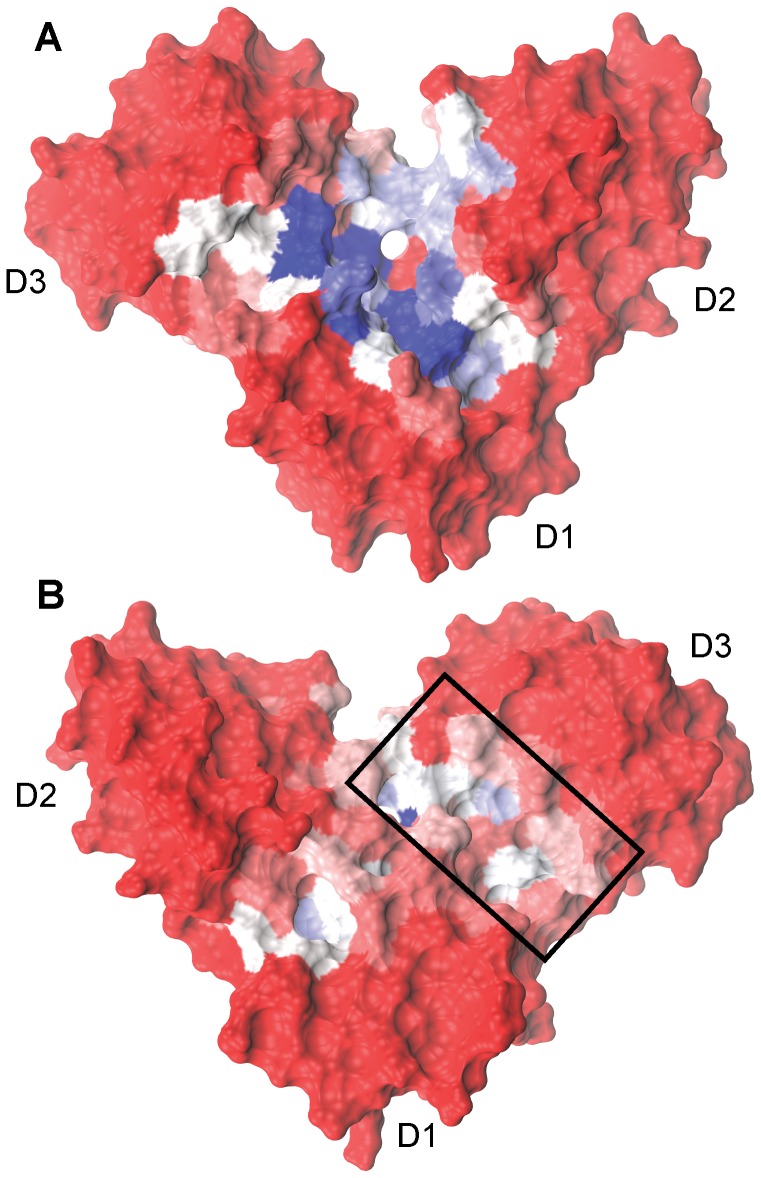
IspH structures as seen from the front (A) and the back (B) with respect to the binding pocket location, colored by normalized FTMAP probe occupancy (red to white to blue follows occupancies of 0.0 to 0.5 to 1.0). (A) illustrates the high propensities of FTMAP fragments to bind to the active site, as well as to the interface between D1 and D3. (B) reveals an allosteric pocket between D1 and D3 (highlighted by the black box).

Taking the fragment positions as they are docked by FTMAP into each representative structure from the [Fe_4_S_4_]^2+^_(open,substrate-free)_ simulations, we synthesize information regarding where the docked fragments congregate by generating a probe occupancy map for IspH. Probe occupancy is highest at the pocket corresponding to the substrate-binding site (Figure 8a, Figure S4). In the more voluminous clusters, as well as the most dominant cluster, probes expand beyond the HMBPP-binding site at the iron, into a crevice between D1 and D3 (Figure 8a, Figure S4). This finding suggests that inhibitors capable of occupying this expanded pocket while locking the protein in a state that is more open with respect to the [Fe_3_S_4_]^+^_(open, substrate-free)_ IspH crystal structure may provide a feasible route toward novel inhibitor design.

An unanticipated finding from solvent mapping concerns the side of IspH opposite the substrate-binding pocket. When the protein opens fully, as seen in the [Fe_4_S_4_]^2+^_(open,substrate-free)_ simulations, the hinge-like quality of the interface between D3 and D1/D2 hyperextends, creating a druggable pocket found opposite the side of the HMBPP-binding site (Figure 8b, black rectangle; Figure S4). When the hinge is opened, this pocket occupies a POVME-measured volume of 330–500 Å^3^ and accommodates a variety of polar and nonpolar probes. This result, stemming from the opening motions intrinsic to substrate-free, [Fe_4_S_4_]^2+^ IspH, may provide an allosteric target for inhibitor design.

## Discussion

Application of the aMD method to sample conformational space in both [Fe_4_S_4_]^2+^_(open,substrate-free)_ and [Fe_4_S_4_]^2+^/HMBPP_(closed)_ states of IspH increases our understanding of how HMBPP binding affects IspH structure and dynamics, as well as highlights alternative routes for the design of novel IspH inhibitors.

In regard to IspH dynamics, our [Fe_4_S_4_]^2+^/HMBPP_(open,docked)_ aMD simulations are able to capture the closing event that accompanies ligand binding in two out of three simulations. In these simulations, residues in D1 that are needed to coordinate HMBPP (His-42 and His-74) are already properly positioned to interact with the pyrophosphate tail of HMBPP, whereas residues in D2 and D3 that coordinate HMBPP require domain motions to bring them in proximity of the substrate. Once D2 and D3 close around HMBPP, it is apparent from our PCA that IspH is unable to reopen, with the fluctuations of residues from D2 and D3 largely suppressed as these domains engage in multiple electrostatic and hydrogen bond interactions with HMBPP (*e.g.* His-124, and Ser-226). These observations underscore the suggestions by others that both electron addition to the substrate and changes in active site and substrate titration states are necessary, not only for catalysis, but also to alter the electrostatics in the active site to enable IspH opening and release of the catalytic product, IPP or DMAPP [24].

In contrast with the [Fe_4_S_4_]^2+^/HMBPP_(open,docked)_ and [Fe_4_S_4_]^2+^/HMBPP_(closed)_ systems, [Fe_4_S_4_]^2+^_(open,substrate-free)_ IspH is much more flexible and thus able to access both closed states and conformations that open beyond what is seen in the [Fe_3_S_4_]^+^_(open, substrate-free)_ IspH crystal structure. When closed in our simulations, projections of [Fe_4_S_4_]^2+^_(open,substrate-free)_ IspH onto PC space show no overlap with the [Fe_4_S_4_]^2+^_(closed, HMBPP-bound)_ IspH crystal structure, indicating that substrate binding allows IspH to sample a closed state that is inaccessible in the absence of HMBPP.

In our simulations of the substrate-free state, IspH accesses both open and closed conformations. Since closed states preexist in the substrate-free ensemble, it is tempting to suggest that conformational selection (CS) [49] is responsible for ligand recognition in IspH. Following the logic of Sullivan and Holyoak [50], however, induced fit (IF) likely better describes the conformational changes occurring upon ligand binding since HMBPP cannot actually bind to the closed state that preexists in substrate-free IspH (due to occlusion of the active site by D2 and D3) [51]. We propose that ligand binding may still be described as a combination of CS and IF, where the ligand initially selects open conformations for formation of an encounter complex. Once initially bound, HMBPP induces closure of D2 and D3 via motions that are also intrinsic to IspH in the absence of ligand. This ligand recognition mechanism, drawing from both CS and IF, is not unique to IspH, but rather gives further support to the suggestions of others that ligand binding can contain elements of both CS and IF [52]–[54].

Returning to the structures observed in the [Fe_4_S_4_]^2+^_(open,substrate-free)_ IspH aMD simulations, it is interesting that the active site volume is subject to significant fluctuations—largely due to the flexibility of the loop regions connecting D3 to D1 and D2 and, to a lesser extent, D2 to D1 and D3. These fluctuations are expected, as HMBPP likely binds initially to an enlarged binding pocket that may accommodate the expansive hydration shell expected for pyrophosphate-containing molecules [55] that HMBPP carries from solution into an encounter complex with IspH. The larger pocket stemming from the super-open state seen in the [Fe_4_S_4_]^2+^_(open,substrate-free)_ simulations would allow for this initial complex to form. Given the presence of these larger pockets in our simulations and this mechanistic rationale, it is reasonable to hypothesize that a variety of differently sized ligands may also be accommodated in the binding pocket.

Combining these volume data for the [Fe_4_S_4_]^2+^_(open,substrate-free)_ state with the results from our KL divergence analysis and FTMAP solvent mapping of IspH, we can build on the work of others [29]–[34] in suggesting a novel framework for future IspH inhibitor design. HMBPP binding to IspH can be regarded the first step in the catalytic process vital to most microbes for production of IPP and DMAPP. Preventing this binding event is thus the goal of competitive inhibitor development.

From our KL divergence analysis, we find that in addition to conserved residues that coordinate HMBPP upon its binding, residues that are adjacent to thiolate residues achieve high “mutual divergence” scores due to their distinct dihedral distributions when IspH is open and closed. Given its position adjacent to the fully conserved Cys-193, Asn-194 likely coordinates the hinge motions of D3 that give way to the necessary closing events that accompany HMBPP binding. Preventing the closing of the D3 hinge and, consequently, locking the Asn-194 backbone dihedrals in their disordered, open conformations may provide a novel mode of inhibiting IspH.

From our aMD simulations, two differing mechanisms for disrupting the hinge motions of D3 are apparent. The first targets the outward motion of D3 from the HMBPP binding site that creates an enlarged cavity that extends from the active site to the interface between D3 and D1 (Figure 8a). Either design of larger competitive inhibitors that interact with the apical iron and the D3/D1 interface or design of ligands that interact allosterically with the D3/D1 interface could successfully exploit the enlarged pocket on the active site side of IspH. Alternatively, the presence of an allosteric pocket opposite the side of the HMBPP binding site may be targeted for inhibitor design (Figure 8b, Figure S4). Both these proposed sites for inhibitor design are “hot spots” found by solvent probes with FTMAP. Noting that probe occupancy correlates well with sequence conservation as measured by Shannon entropy (r = 0.49) provides further support for these suggested modes of inhibition.

Given the documented difficulties of rational drug design for metalloproteins, notably from a computational perspective [56], allosteric sites that do not require a detailed description of metal binding (*e.g.* orbital interactions, polarization and charge transfer) are highly desirable if existent. Furthermore, it has been shown that perturbations to allosteric networks in redox-active metalloproteins may affect the redox potential of these proteins and, consequently, alter their activities [57]. These factors motivate us to include the different pockets revealed by aMD simulations, particularly those that may provide routes to noncompetitive inhibition, in future computer aided drug design workflows.

### Conclusion

Using aMD simulations, we are able to capture the closing event that accompanies the binding of HMBPP to IspH when starting from the substrate-free crystal structure. Drawing from PCA and visual analyses of the different trajectories considered, we propose that ligand binding occurs via a combination of induced fit and conformational selection. We note that a single dihedral angle, the ψ angle in Asn-194, selects for either open or closed conformations of IspH, the latter being achieved via a crank motion that draws D3 inward to corral the active site. Furthermore, our aMD simulations reveal both an expanded active site pocket encompassing a crevice between D1 and D3, as well as an allosteric pocket between D1 and D3 on the side opposite the substrate binding pocket that may be utilized for the design of novel IspH inhibitors.

## Methods

### Ligand parameterization for molecular dynamics simulation

Since the questions under consideration in this study begin with open, substrate-free IspH protein, we use the [Fe_3_S_4_]^+^_(open, substrate-free)_ IspH crystal structure from Rekittke, *et al* (PDB ID: 3DNF) as a starting point [23]. Applying insight from the [Fe_4_S_4_]^2+^_(closed, HMBPP-bound)_ IspH crystal structure from Gräwert, *et al.* (PDB ID: 3KE8) [28], we model the apical iron into the cluster by superposition.

Using the Amsterdam Density Functional program [58], a model [Fe_4_S_4_]^2+^ cluster is geometry optimized using broken symmetry density functional theory (BS-DFT) [59], [60] at the OLYP/TZP level of theory [61], [62]. With the Gaussian 09 suite of programs [63], we optimize the geometry of HMBPP and compute the electrostatic potentials of both geometry optimized HMBPP and the model [Fe_4_S_4_]^2+^ cluster using MK radii [64] at the HF/6-31G(d) level of theory. The antechamber program [65] in the AmberTools 13 suite of programs [66] is then used to apply the restrained electrostatic potential (RESP) procedure to derive point charges for use in MD simulations. In the case of the [Fe_4_S_4_]^2+^ cluster, parameters for nonbonded terms are taken from the AMBER GAFF force field [67], and bonds and angles between atoms are implicitly accounted for by harmonic restraints applied to these terms, using parameters from the [Fe_4_S_4_]^2+^_(closed, HMBPP-bound)_ IspH crystal structure [28]. For HMBPP, all force field parameters are taken from the AMBER GAFF force field [67]. All charge and nonbonded parameters, as well as, a more detailed discussion of the ligand parameterization process, are included in the Supporting Information.

### System preparation for production molecular dynamics simulations

Hydrogens are added using PDB2PQR [68], [69], with protonation states assigned using the PROpKa program [70]. In our setup, His-42 and His-124 are set to their imidazolium states, and Glu-126 is protonated. Following hydrogen addition, the protein systems are minimized for 2000 steps in the gas phase using the sander module in AMBER12 [66], to remove problematic steric clashes. The systems are solvated in a box of TIP3P waters [71] that extends 12 Å beyond the closest solute atom, with counterions added to enforce electroneutrality. Non-water bonds to hydrogen atoms are constrained using the SHAKE algorithm [72], while the O-H bonds in water are constrained using the SETTLE algorithm [73]. All protein force field parameters are taken from the AMBER ff99SB force field [74], while the ligand parameters referred to above are taken from the AMBER GAFF force field [67]. Subsequent 2000 step minimizations are performed (a) to relax the water with protein fixed by positional constraints, (b) to relax the protein with all waters constrained, and (c) relax the whole system. Following this minimization protocol, all systems are equilibrated at constant pressure and temperature (NPT) conditions for 1 ns, with the protein fixed by positional constraints. The pressure is regulated using the Berendsen barostat [75] with isotropic position scaling (ntp = 1) and a pressure relaxation time of 2.0 ps, while a Langevin thermostat [76] with collision frequency of 2.0 ps^−1^ is used to increase the temperature of the system from 0 to 300K. The protein constraints are then lifted and a subsequent 2 ns NPT equilibration is performed at 300K to verify the density of the system is reasonable and stable. The last equilibration step is performed at constant volume and temperature (NVT) for 5 ns at 300K to prepare the system for production MD simulations. All dynamics are conducted using the pmemd.cuda engine [66], [77], with Particle Mesh Ewald summations used for computing long-range electrostatic interactions and short-range nonbonded interactions truncated beyond a cutoff of 10 Å [78], [79].

### Accelerated molecular dynamics (aMD) simulations of IspH

Given current computational power, most MD simulations are limited to sampling timescales on the order of 10–1000 ns. Since many biomolecular processes, including, for example, protein folding, ligand binding, and cis/trans isomerization events, may occur on the order of milliseconds to days, enhanced sampling techniques that facilitate traversing of configuration space efficiently are often implemented to provide information about the relevant conformations of biomolecules [80], [81]. Accelerated molecular dynamics (aMD) simulations promote enhanced sampling of systems without the need for defining a reaction coordinate. In aMD simulations, when the potential energy of the system, V(r), is below a threshold energy level, E, a boost energy, ΔV(r), is applied to encourage exploration of other areas of phase space (Eq. 1). The parameter α modulates the aggressiveness of this boost by altering the depth of the wells in the modified potential.
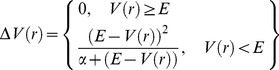
(1)


We employ the dual-boost implementation of aMD to boost both dihedral and total potential energy force field terms to promote side chain dihedral angle rotations and diffusive transitions, respectively [82], [83]. We set the parameters E and α for our systems by defining these variables for the dihedral and total potential energy components with respect to the number of residues in the system, N_res_, and the number of atoms in the system, N_atoms_, respectively (Eq. 2–5):
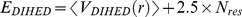
(2)

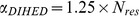
(3)

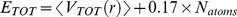
(4)

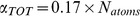
(5)


Subsequent reweighting of the trajectory frames from the aMD simulations using a tenth-order Maclaurin series expansion allows us to extract canonical ensemble averages of the system (further details included in Text S2). Recently, both these methodologies for obtaining aMD parameters and reweighting aMD results were successfully applied to bovine pancreatic trypsin inhibitor (BPTI) to properly obtain the relative populations of relevant, low-lying energetic states [84]. For some semblance of statistics, 3×100 ns aMD simulations are performed on all systems explored in this study.

### Molecular dynamics analysis

RMSD, RMSF, clustering, and dihedral angle analyses are all performed using the AmberTools 12 suite of programs [66]. Alignment procedures implemented in the RMSD and tRMSF calculations are performed with respect to the [Fe_3_S_4_]^+^_(open, substrate-free)_ IspH crystal structure (PDB ID: 3DNF [23]), aligning to the backbone atoms of D1, as this domain is the most rigid in all simulations. Clustering analyses for each of the simulations use pairwise RMSD computed for C_α_ atoms between frames to divide the cumulative trajectories for each system simulated into eight clusters using the average-linkage algorithm [85].

### Principal component analysis

Principal component analysis (PCA) reduces atomic fluctuations in the various trajectories into vectors that represent the dominant correlated motions present in the simulations [86], [87]. Since we want our PCA to assess how well the different simulations sample conformational space with respect to the [Fe_3_S_4_]^+^_(open, substrate-free)_ and [Fe_4_S_4_]^2+^_(closed, HMBPP-bound)_ IspH crystal structures (PDB ID: 3DNF and 3KE8, respectively), we first align the two crystal structures using the STructural Alignment of Multiple Proteins (STAMP) procedure [88], as implemented in the VMD MultiSeq plugin [89], [90]. The indices of aligned residues are then used in subsequent PCA.

Principal component (PC) space is constructed from the three [Fe_4_S_4_]^2+^_(open,substrate-free)_ simulations and three [Fe_4_S_4_]^2+^/HMBPP_(open,docked)_ simulations. The trajectories for each set of simulations ([Fe_4_S_4_]^2+^_(open,substrate-free)_, [Fe_4_S_4_]^2+^/tHMBPP_(open,docked)_, [Fe_4_S_4_]^2+^/HMBPP_(closed)_) are then projected onto the first and second principal components. Additionally, [Fe_3_S_4_]^+^_(open, substrate-free)_ and [Fe_4_S_4_]^2+^_(closed, HMBPP-bound)_ IspH crystal structures are projected onto PC space to assess overlap between the simulations and these structures along the PC1 and PC2 coordinates. The modes that correspond to PC1 and PC2 are visualized using the Bio3D suite of programs [40].

### Comparing dihedral angle distributions using Kullback-Leibler Divergence

We quantify differences in IspH structure upon ligand binding by applying the Kullback-Leibler (KL) Divergence expansion, also referred to as relative entropy, to assess differences in the distributions of φ, ψ, and χ dihedral angles in [Fe_4_S_4_]^2+^_(open,substrate-free)_ and [Fe_4_S_4_]^2+^/HMBPP_(closed)_ ensembles generated by aMD simulations. To obtain the KL divergence for each residue, we first split the 3×100 ns sets of simulations for the [Fe_4_S_4_]^2+^_(open,substrate-free)_ and [Fe_4_S_4_]^2+^/HMBPP_(closed)_ systems into 6 sets of 50 ns to provide statistical robustness to the calculations. The MutInf program [42], [44] processes the dihedral angle distributions for each of these 50 ns blocks as computed by the g_torsion program from the GROMACS suite of programs [91], computing the KL Divergence for a specific dihedral angle using Eq. 6:
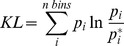
(6)


In this equation, p_i_ refers to the probability that a particular dihedral angle from the [Fe_4_S_4_]^2+^/HMBPP_(closed)_ simulations falls into a specific range of torsional space, which has been divided into 12° bins. The term p_i_* is the corresponding probability that the same dihedral angle from the [Fe_4_S_4_]^2+^_(open,substrate-free)_ simulation falls into the same bin. Combining the KL terms for each of the dihedral angles (φ, ψ, and χ's) of a given residue provides a value for the KL divergence of a specific residue:
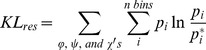
(7)


This value for the KL divergence of a given residue provides a measure of the difference between the dihedral angle probability distribution functions of the [Fe_4_S_4_]^2+^/HMBPP_(closed)_ simulations with respect to the [Fe_4_S_4_]^2+^_(open,substrate-free)_ reference simulations.

### Sequence conservation

Using the Bio3D suite of programs, we compute the Shannon entropy [45], [46] according to equation 3 for all residues in the *A. aeolicus* IspH structure with a 22-letter alphabet, where the 20 amino acids are included, as well as a gap character ‘-’ and a mask character ‘X’ [40].
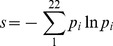
(8)


After normalizing the Shannon entropy score, residues that are fully conserved assume the value 1, while residues with no conservation have a Shannon entropy of 0.

### FTMAP

We employ FTMAP [48] to allow many drug-like, organic fragments to bind to representative structures from the dominant clusters from [Fe_4_S_4_]^2+^_(open,substrate-free)_ aMD simulations. FTMAP utilizes a fast Fourier transform (FFT) algorithm to allow the organic probes to sample many positions along the protein surface. Prevalence of fragment hits along the protein surface signifies “hot spots” that correspond to potentially druggable pockets [48].

We measure the ability of residues in substrate-free IspH to bind FTMAP probes by first defining binding of the residue by the probe as existent if the distance between their respective heavy atoms is less than 5 Å. We then combine these binding results across all dominant clusters from the [Fe_4_S_4_]^2+^_(open,substrate-free)_ aMD simulations and count the number of probes that bind each residue. Normalization of these data indicates the relative propensity of each residue to bind drug-like molecules [92].

## Supporting Information

Figure S1 Plots of RMSD relative to the [Fe_4_S_4_]^2+^_(closed, HMBPP-bound)_ IspH crystal structure (PDB ID: 3KE8, ref. 28 in the Text) over the course of 3×100 ns aMD simulations of (A) [Fe_4_S_4_]^2+^_(open,substrate-free)_, (B) [Fe_4_S_4_]^2+^/HMBPP_(open,docked)_, and (C) [Fe_4_S_4_]^2+^/HMBPP_(closed)_ IspH.
(TIF)

Figure S2 Visualization of IspH motions along PC1 from (A) head-on toward the binding site and (B) from a top-view. As the principal components are constructed from both [Fe_4_S_4_]^2+^_(open,substrate-free)_ and [Fe_4_S_4_]^2+^/HMBPP_(open,docked)_ simulations, the dominant motion is the super-opening to closing of D3, with smaller closing motions of D2.
(TIF)

Figure S3 Distributions of φ and ψ angles for Phe-12, Thr-95 and Asn-194 (the three having “mutual divergence” of 3.06, 0.77 and 0.32, respectively). (A) φ angle distributions in [Fe_4_S_4_]^2+^/HMBPP_(open,docked)_ simulations; (B) φ angle distributions in [Fe_4_S_4_]^2+^/HMBPP_(closed)_ simulations; (C) ψ angle distributions in [Fe_4_S_4_]^2+^/HMBPP_(open,docked)_ simulations; (D) ψ angle distributions in [Fe_4_S_4_]^2+^/HMBPP_(closed)_ simulations.
(TIF)

Figure S4 (A) Plot of normalized FTMAP probe occupancy with respect to individual residues of IspH. Probes binding to the expanded substrate binding pocket (B) are marked by black stars, whereas probes that stick to the allosteric site (C), opposite the side of the substrate binding site, are marked by red stars.
(TIF)

Figure S5 Visual representation of the [Fe_4_S_4_(SCH_3_)_3_OH_2_]^1−^ model cluster utilized to obtain charges for the [4Fe-4S]^2+^ cluster and its coordinating thiolate residues. Atom labels correspond to those accompanying charges in Table S1.
(TIF)

Figure S6 Atom labels that correspond to the charges and atom types for the HMBPP molecule given in Table S3.
(TIF)

Table S1 Charge parameters for the [4Fe-4S]^2+^ cluster and its liganded cysteines.
(PDF)

Table S2 Nonbonded parameters used for the [4Fe-4S]^2+^ cluster in simulations of IspH (references 12 and 13 in Text S1).
(PDF)

Table S3 Force field parameters used for HMBPP. The atom types listed are assigned their respective nonbonded parameters in the AMBER GAFF force field [67].
(PDF)

Text S1 Procedure for obtaining force field parameters for the [4Fe-4S] cluster, its coordinating cysteines and HMBPP.
(PDF)

Text S2 Procedure employed for reweighting aMD trajectories in the principal component analyses.
(PDF)
