# Supplementary figures and images for: Utilizing a Dynamical Description of IspH to Aid in the Development of Novel Antimicrobial Drugs
Source: PLoS Comput Biol. 2013 Dec 19;9(12):e1003395. doi: 10.1371/journal.pcbi.1003395 (PMC3868525; doi:10.1371/journal.pcbi.1003395)

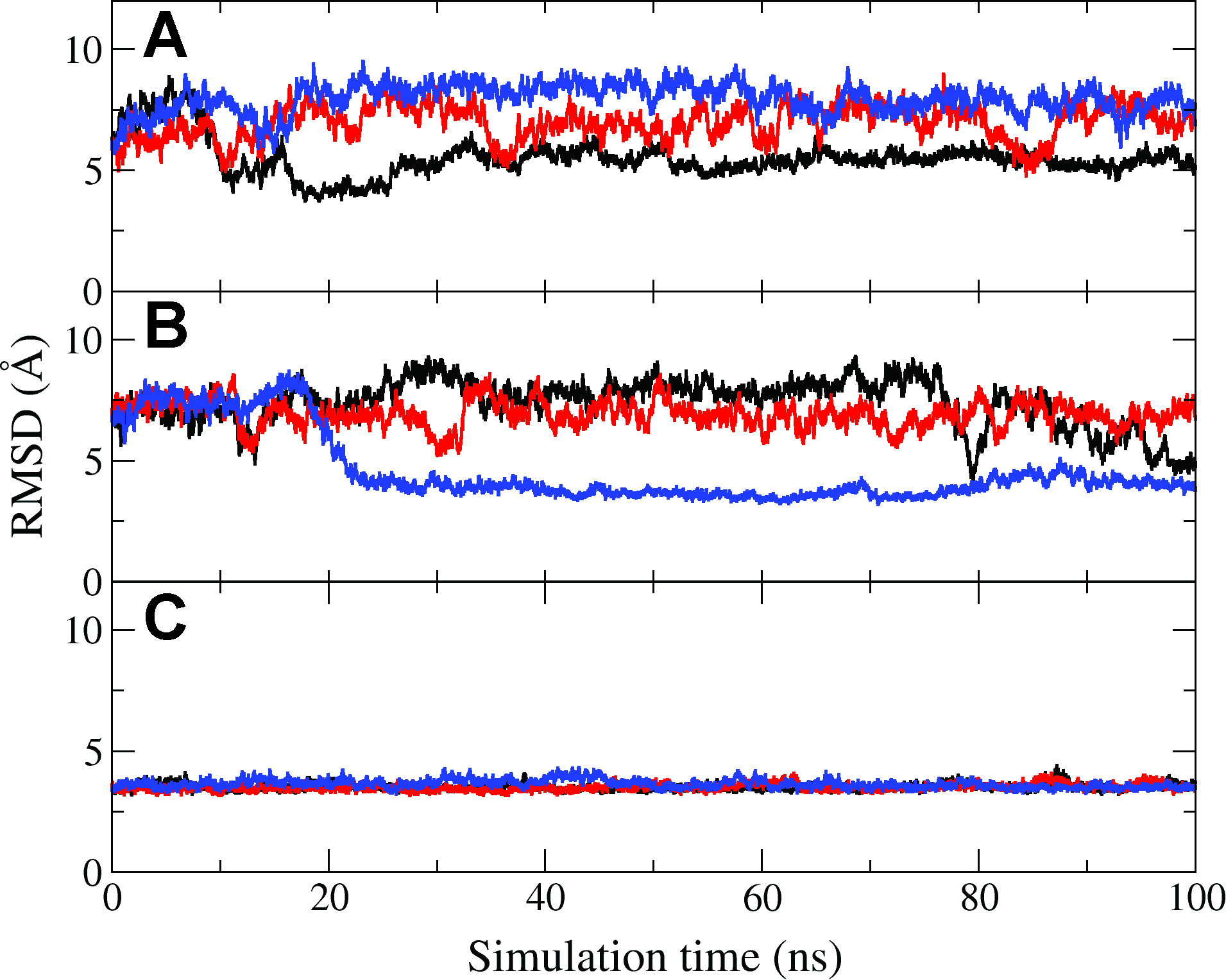

Supplement: Figure S1 — Plots of RMSD relative to the [Fe4S4]2+ (closed, HMBPP-bound) IspH crystal structure (PDB ID: 3KE8, ref. 28 in the Text) over the course of 3×100 ns aMD simulations of (A) [Fe4S4]2+ (open,substrate-free), (B) [Fe4S4]2+/HMBPP(open,docked), and (C) [Fe4S4]2+/HMBPP(closed) IspH. (TIF) [file pcbi.1003395.s001.tif]

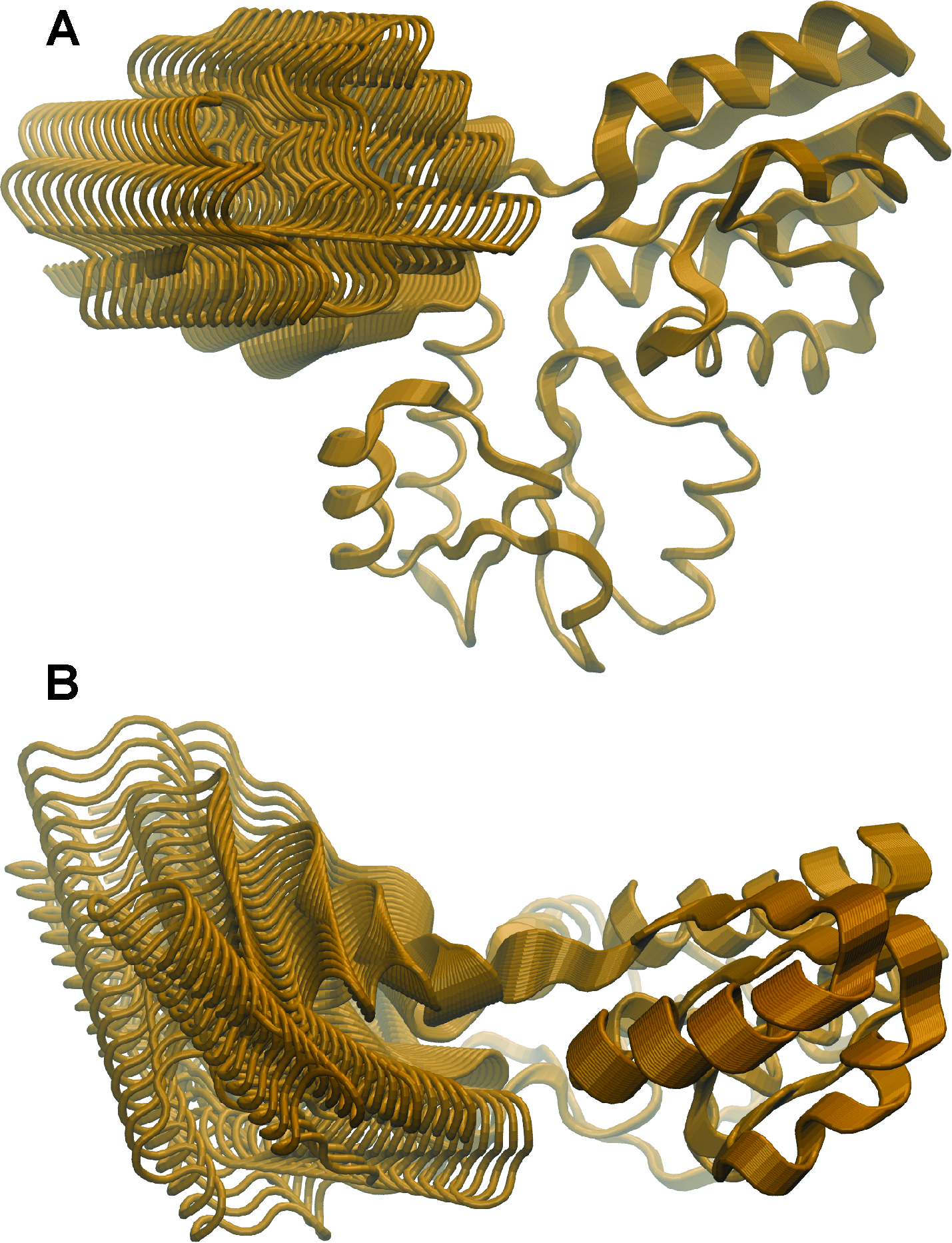

Supplement: Figure S2 — Visualization of IspH motions along PC1 from (A) head-on toward the binding site and (B) from a top-view. As the principal components are constructed from both [Fe4S4]2+ (open,substrate-free) and [Fe4S4]2+/HMBPP(open,docked) simulations, the dominant motion is the super-opening to closing of D3, with smaller closing motions of D2. (TIF) [file pcbi.1003395.s002.tif]

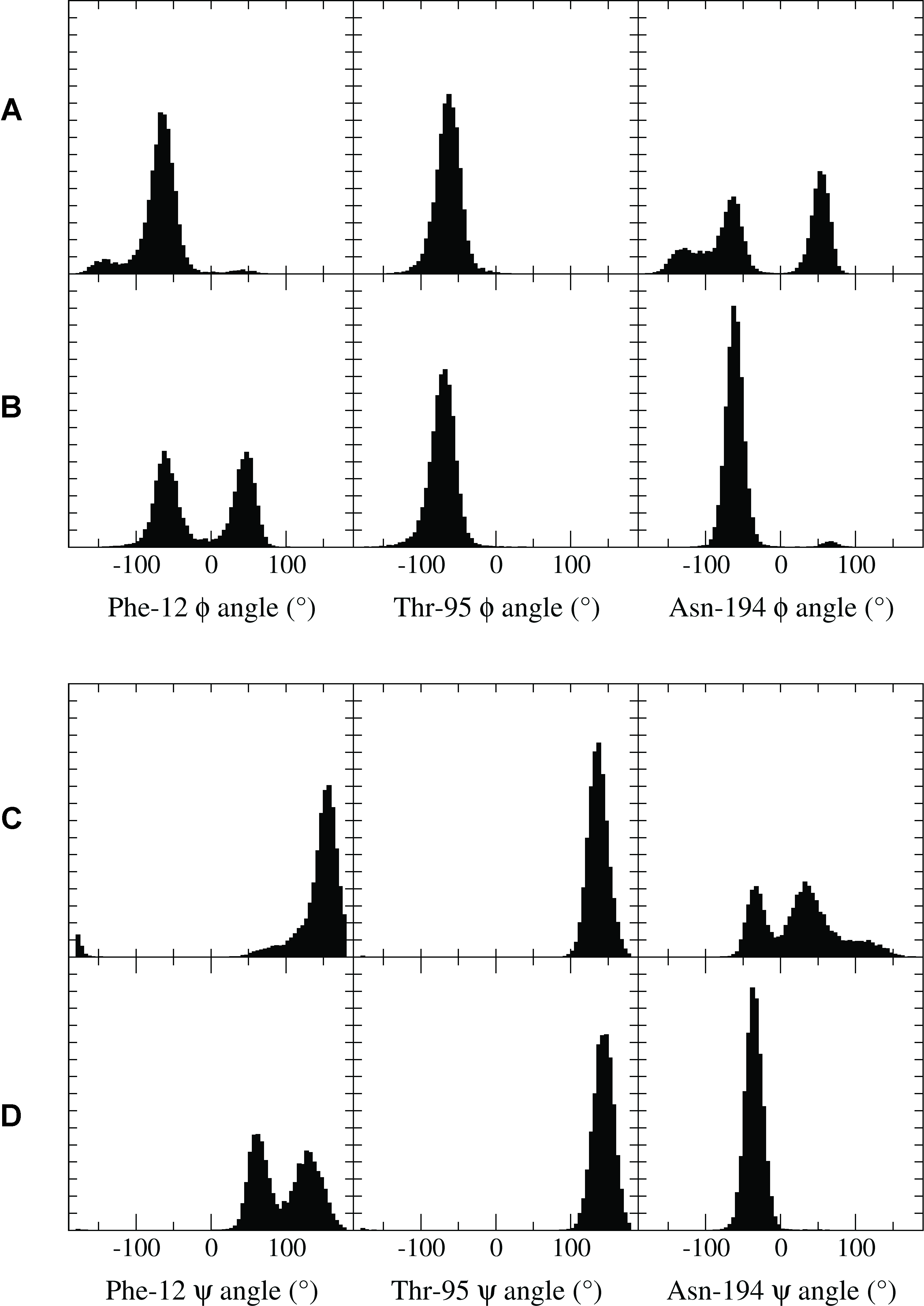

Supplement: Figure S3 — Distributions of φ and ψ angles for Phe-12, Thr-95 and Asn-194 (the three having “mutual divergence” of 3.06, 0.77 and 0.32, respectively). (A) φ angle distributions in [Fe4S4]2+/HMBPP(open,docked) simulations; (B) φ angle distributions in [Fe4S4]2+/HMBPP(closed) simulations; (C) ψ angle distributions in [Fe4S4]2+/HMBPP(open,docked) simulations; (D) ψ angle distributions in [Fe4S4]2+/HMBPP(closed) simulations. (TIF) [file pcbi.1003395.s003.tif]

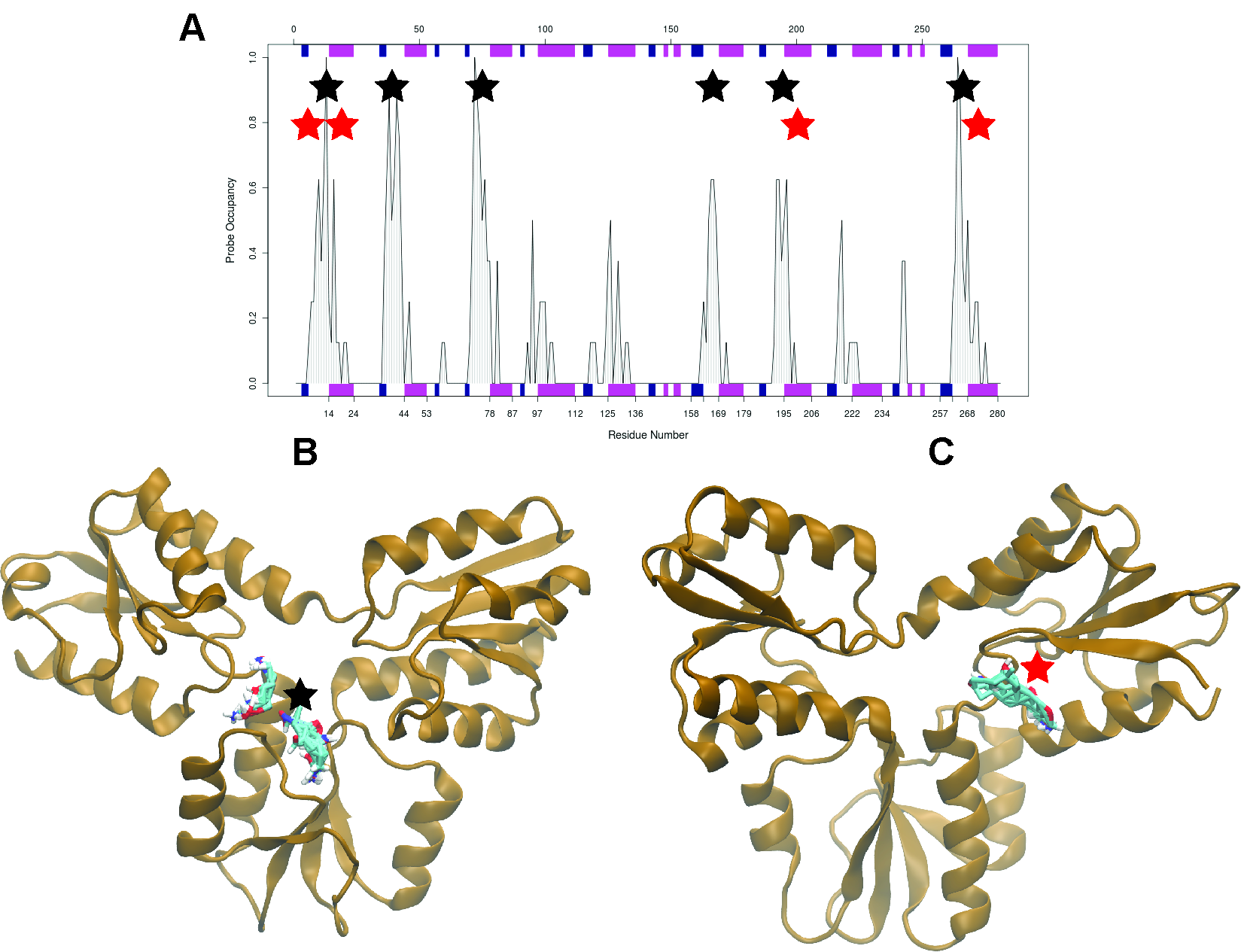

Supplement: Figure S4 — (A) Plot of normalized FTMAP probe occupancy with respect to individual residues of IspH. Probes binding to the expanded substrate binding pocket (B) are marked by black stars, whereas probes that stick to the allosteric site (C), opposite the side of the substrate binding site, are marked by red stars. (TIF) [file pcbi.1003395.s004.tif]

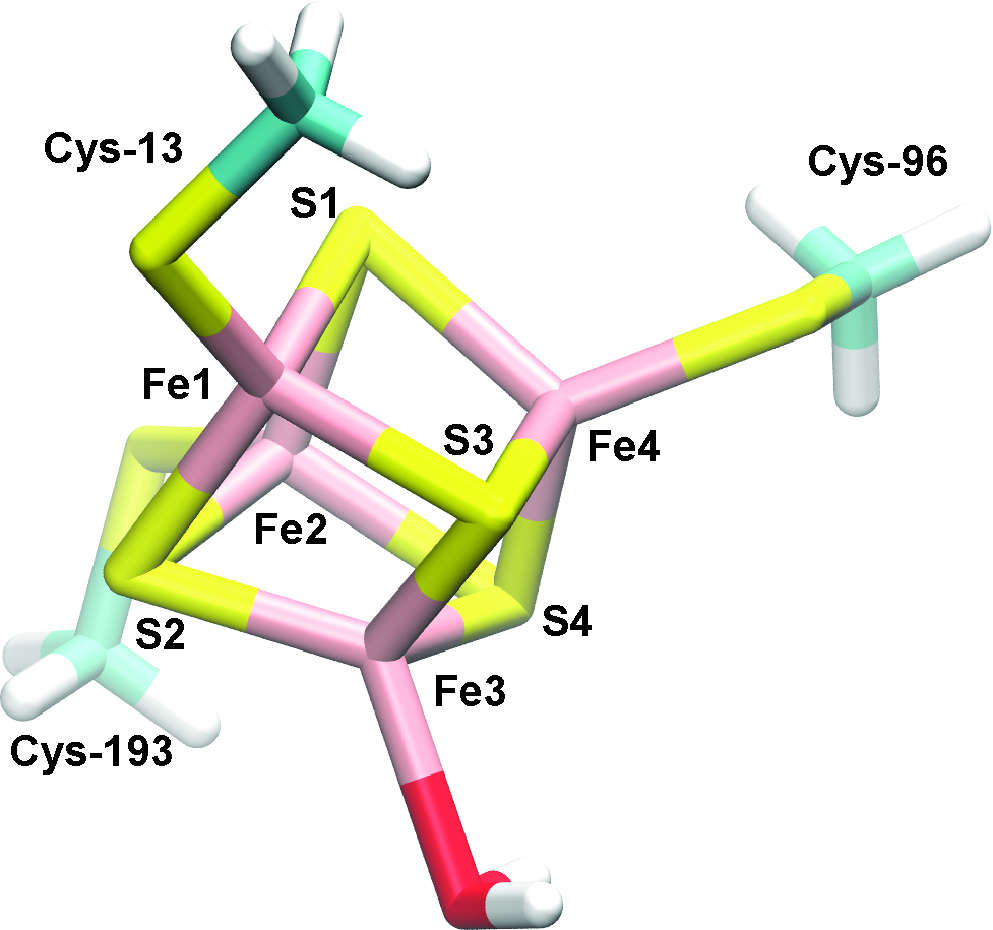

Supplement: Figure S5 — Visual representation of the [Fe4S4(SCH3)3OH2]1− model cluster utilized to obtain charges for the [4Fe-4S]2+ cluster and its coordinating thiolate residues. Atom labels correspond to those accompanying charges in Table S1. (TIF) [file pcbi.1003395.s005.tif]

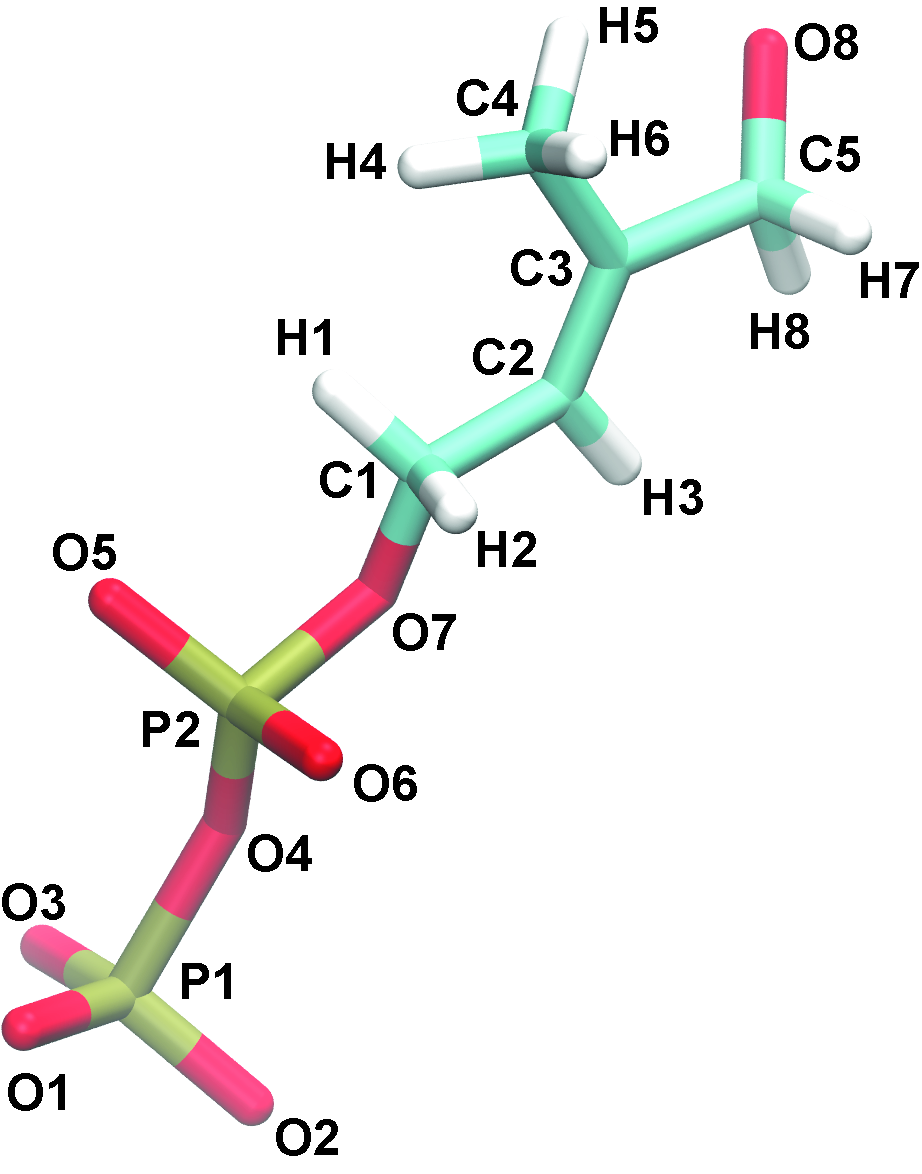

Supplement: Figure S6 — Atom labels that correspond to the charges and atom types for the HMBPP molecule given in Table S3. (TIF) [file pcbi.1003395.s006.tif]
