# Supplementary material for: Utilizing a Dynamical Description of IspH to Aid in the Development of Novel Antimicrobial Drugs
Source: PLoS Comput Biol. 2013 Dec 19;9(12):e1003395. doi: 10.1371/journal.pcbi.1003395 (PMC3868525; doi:10.1371/journal.pcbi.1003395)
Supplement: Table S1 — Charge parameters for the [4Fe-4S]2+ cluster and its liganded cysteines. (PDF) [file pcbi.1003395.s007.pdf]

Table S1. Charge parameters for $[4Fe4S]^{2+}$ cluster and its liganded cysteines. The net charge for the cluster plus its three coordinating thiolates is -1.

[4Fe-4S]	
Fe1	0.703
Fe2	0.772
Fe3	0.981
Fe4	0.652
S1	-0.447
S2	-0.738
S3	-0.736
S4	-0.788

	CYS13	CYS96	CYS193
N	-0.463	-0.463	-0.463
HN	0.252	0.252	0.252
CA	0.136	0.145	0.143
HA	0.048	0.048	0.048
CB	-0.177	-0.177	-0.177
HB3	0.085	0.094	0.092
HB2	0.085	0.094	0.092
SG	-0.677	-0.529	-0.477
C	0.616	0.616	0.616
O	-0.504	-0.504	-0.504
