# Supplementary material for: Utilizing a Dynamical Description of IspH to Aid in the Development of Novel Antimicrobial Drugs
Source: PLoS Comput Biol. 2013 Dec 19;9(12):e1003395. doi: 10.1371/journal.pcbi.1003395 (PMC3868525; doi:10.1371/journal.pcbi.1003395)
Supplement: Table S2 — Nonbonded parameters used for the [4Fe-4S]2+ cluster in simulations of IspH (references 12 and 13 in Text S1). (PDF) [file pcbi.1003395.s008.pdf]

Table S2. Nonbonded parameters used in simulations of IspH (taken from ref. 12 and 13 in Text S1).

	r	ε
Fe	1.20	0.05
S	2.00	0.25
