# Supplementary material for: Utilizing a Dynamical Description of IspH to Aid in the Development of Novel Antimicrobial Drugs
Source: PLoS Comput Biol. 2013 Dec 19;9(12):e1003395. doi: 10.1371/journal.pcbi.1003395 (PMC3868525; doi:10.1371/journal.pcbi.1003395)
Supplement: Table S3 — Force field parameters used for HMBPP. The atom types listed are assigned their respective nonbonded parameters in the AMBER GAFF force field [67]. (PDF) [file pcbi.1003395.s009.pdf]

Table S3. Force field parameters for HMBPP.

Atom Name	Charge	AMBER GAFF Atom type
O1	-0.905	o
O2	-0.905	o
O3	-0.905	o
P1	1.206	p5
O4	-0.568	os
O5	-0.893	o
O6	-0.893	o
P2	1.345	p5
O7	-0.651	os
C1	0.493	c3
H1	-0.056	h1
H2	-0.056	h1
C2	-0.408	c2
H3	0.128	ha
C3	0.009	c2
C4	-0.101	c3
H4	-0.004	hc
H5	-0.004	hc
H6	-0.004	hc
C5	0.563	c3
H7	-0.214	h1
H8	-0.214	h1
O8	-0.963	o
