# Supplementary material for: Utilizing a Dynamical Description of IspH to Aid in the Development of Novel Antimicrobial Drugs
Source: PLoS Comput Biol. 2013 Dec 19;9(12):e1003395. doi: 10.1371/journal.pcbi.1003395 (PMC3868525; doi:10.1371/journal.pcbi.1003395)
Supplement: Text S1 — Procedure for obtaining force field parameters for the [4Fe-4S] cluster, its coordinating cysteines and HMBPP. (PDF) [file pcbi.1003395.s010.pdf]

Text S1.

Force field parameters for the [4Fe4S] cluster, its coordinating cysteines and HMBPP

To obtain charges for the [4Fe4S] cluster, as well as the three thiolate residues that coordinate it, we construct a model cluster of the form $[Fe_4S_4(SCH_3)_3OH_2]^{1-}$ (Figure S5). The formal charges of this model cluster are as follows: a spin-coupled ferric/ferrous iron pair (1 x Fe^{3+}; 1 x Fe^{2+}), a spin-coupled, delocalized mixed-valence iron pair (2 x $Fe^{2.5+}$), four inorganic sulfides (4 x S^{2-}) and three methyl thiolates (3 x $^{-}SCH_3$). A water is placed in the model to coordinate the unliganded iron.

The [4Fe4S] model cluster is geometry optimized using the Amsterdam Density Functional program [1-3] with broken symmetry density functional theory (BS-DFT) [4,5]. In practice, this is achieved by following the procedure implemented by Noodleman and co-workers in studying Fe-S clusters, which involves three separate calculations: (1) a high spin (ferromagnetic) single point calculation is performed, with all iron net spins aligned parallel; (2) the spins on one Fe-Fe pair are flipped, such that there are two sets of Fe-Fe pairs containing spins that are oppositely aligned, allowing a broken symmetry single point calculation to be performed; (3) utilizing the broken symmetry wavefunction, the model cluster is geometry optimized [6]. Geometry optimization is achieved using the OLYP functional [7,8] with the TZP basis set. This method has previously been shown to give good

agreement with experimental geometries and spectroscopic parameters for Fe-S systems [9].

Following geometry optimization, the electrostatic potential (ESP) is computed at the HF/6-31G(d) level of theory using the Gaussian 09 suite of programs [10]. From the ESP, charges are obtained using the standard RESP procedure within the AMBER suite of programs [11]. The values of these charges, which correspond to the atom labels shown in Figure S5, are given in Table S1. For the $Fe^{2+/3+}$ and S^{2-} nonbonded parameters, we employ the values obtained and utilized by others for heme groups [12] and thiolates [13], respectively (Table S2).

To parameterize HMBPP, the molecule is first geometry optimized at the B3LYP/6-31G(d) level of theory [8,14-16] using the Gaussian 09 suite of programs [10]. The electrostatic potential is obtained at the HF/6-31G(d) level of theory, from which point charges are extracted with RESP. The charges for all HMBPP atoms are included along with their respective AMBER GAFF force field atom types (Table S3), the latter determining the HMBPP nonbonded parameters used in this work [17]. For reference, atom names given in Table S3 are included in a visual representation of HMBPP in Figure S6.

References

1. Guerra CF, Snijders JG, te Velde G, Baerends EJ (1998) Towards an order-N DFT method. Theoretical Chemistry Accounts 99: 391-403.

2. te Velde G, Bickelhaupt FM, Baerends EJ, Guerra CF, Van Gisbergen SJA, et al. (2001) Chemistry with ADF. Journal of Computational Chemistry 22: 931-967.
3. ADF2009. Amsterdam, The Netherlands: SCM, Theoretical Chemistry, Vrije Universiteit.
4. Noodleman L, Case DA (1992) Density-Functional Theory of Spin Polarization and Spin Coupling in Iron-Sulfur Clusters. Advances in Inorganic Chemistry 38: 423-470.
5. Torres RA, Lovell T, Noodleman L, Case DA (2003) Density functional-and reduction potential calculations of Fe(4)S(4) clusters. Journal of the American Chemical Society 125: 1923-1936.
6. Sandala GM, Noodleman L (2011) Modeling the MoFe nitrogenase system with broken symmetry density functional theory. Methods in molecular biology 766: 293-312.
7. Handy NC, Cohen AJ (2001) Left-right correlation energy. Molecular Physics 99: 403-412.
8. Lee CT, Yang WT, Parr RG (1988) Development of the Colle-Salvetti Correlation-Energy Formula into a Functional of the Electron-Density. Physical Review B 37: 785-789.
9. Noodleman L, Han WG (2006) Structure, redox, pK(a), spin. A golden tetrad for understanding metalloenzyme energetics and reaction pathways. Journal of Biological Inorganic Chemistry 11: 674-694.
10. Frisch MJ, Trucks GW, Schlegel HB, Scuseria GE, Robb MA, et al. (2009) Gaussian 09, Revision A.1. Wallingford, CT: Gaussian, Inc.
11. Case DA, Darden TA, Cheatham III TE, Simmerling CL, Wang J, et al. (2012) AMBER 12. University of California, San Francisco.
12. Giammona D (1984) An examination of conformational flexibility in porphyrins and bulky-ligand binding in myoglobin: University of California, Davis.
13. Hornak V, Abel R, Okur A, Strockbine B, Roitberg A, et al. (2006) Comparison of multiple amber force fields and development of improved protein backbone parameters. Proteins-Structure Function and Bioinformatics 65: 712-725.
14. Becke AD (1993) Density-Functional Thermochemistry .3. The Role of Exact Exchange. Journal of Chemical Physics 98: 5648-5652.
15. Stephens PJ, Devlin FJ, Chabalowski CF, Frisch MJ (1994) Ab-Initio Calculation of Vibrational Absorption and Circular-Dichroism Spectra Using Density-Functional Force-Fields. Journal of Physical Chemistry 98: 11623-11627.
16. Vosko SH, Wilk L, Nusair M (1980) Accurate Spin-Dependent Electron Liquid Correlation Energies for Local Spin-Density Calculations - a Critical Analysis. Canadian Journal of Physics 58: 1200-1211.
17. Wang JM, Wolf RM, Caldwell JW, Kollman PA, Case DA (2004) Development and testing of a general amber force field. Journal of Computational Chemistry 25: 1157-1174.
