# Supplementary material for: Utilizing a Dynamical Description of IspH to Aid in the Development of Novel Antimicrobial Drugs
Source: PLoS Comput Biol. 2013 Dec 19;9(12):e1003395. doi: 10.1371/journal.pcbi.1003395 (PMC3868525; doi:10.1371/journal.pcbi.1003395)
Supplement: Text S2 — Procedure employed for reweighting aMD trajectories in the principal component analyses. (PDF) [file pcbi.1003395.s011.pdf]

**Text S2.**

**Employing reweighted aMD trajectories in the principal component analyses**

The principal component analysis (PCA) plots presented in this work follow the procedure outlined by Pierce *et al.* and employ a 2D histogram across the PC1 and PC2 coordinates [1]. PC space is divided into bins of dimension 1Å x 1Å, and points sampled during the aMD simulations are placed into their respective bins. To facilitate this procedure computationally, we utilize an indicator function, $\delta_{k,ij}$, that accounts for whether each point in PC space, (PC1, PC2), falls into the bin ($PC1_i$, $PC2_j$) for a given trajectory frame, k (Eq. 1).

$$(1)\quad \delta_{k,ij} = \begin{Bmatrix} 1, & (PC1, PC2)_k \in (PC1_i, PC2_j) \\ 0, & otherwise \end{Bmatrix}$$

Using $\delta_{k,ij}$, the histogram at bin ($PC1_i$, $PC2_j$) can be reweighted using Eq. 2, where K is the total number of trajectory frames and $\Delta V_k$ is the total boost potential applied at frame k.

$$(2)\quad H_{ij} = \sum_{k=1}^{K} \delta_{k,ij} * \exp\left(\beta \Delta V_k\right)$$

In our analyses, we perform this reweighting using a tenth-order Maclaurin series expansion of the exponential (Eq. 3).

$$(3)\quad \exp(\beta \Delta V_k) \approx 1 + \frac{\beta \Delta V_k}{1!} + \frac{(\beta \Delta V_k)^2}{2!} + \frac{(\beta \Delta V_k)^3}{3!} + \ldots + \frac{(\beta \Delta V_k)^{10}}{10!}$$

After reweighting all the histograms in PC space, we obtain the PC1-PC2 free energy plot shown in Figure 5 using Eq. 4,

$$(4)\quad W_{ij} = -k_B T \ln H_{ij} + W_0 \;,$$

where $k_B$ is the Boltzmann constant, T is temperature (taken to be 300K) and $W_0$ is a constant chosen to set the free energy minimum to zero.

**References**


1. Pierce LCT, Salomon-Ferrer R, de Oliveira CAF, McCammon JA, Walker RC (2012) Routine Access to Millisecond Time Scale Events with Accelerated Molecular Dynamics. Journal of Chemical Theory and Computation 8: 2997-3002.
